# The Wound Microenvironment Reprograms Schwann Cells to Invasive Mesenchymal-like Cells to Drive Peripheral Nerve Regeneration

**DOI:** 10.1016/j.neuron.2017.09.008

**Published:** 2017-09-27

**Authors:** Melanie P. Clements, Elizabeth Byrne, Luis F. Camarillo Guerrero, Anne-Laure Cattin, Leila Zakka, Azhaar Ashraf, Jemima J. Burden, Sanjay Khadayate, Alison C. Lloyd, Samuel Marguerat, Simona Parrinello

**Affiliations:** 1 Cell Interactions and Cancer Group, MRC London Institute of Medical Sciences, Du Cane Road, London W12 0NN, United Kingdom; 2 Institute of Clinical Sciences, Faculty of Medicine, Imperial College London, Du Cane Road, London W12 0NN, United Kingdom; 3 Quantitative Gene Expression Group, MRC London Institute of Medical Sciences, Du Cane Road, London W12 0NN, United Kingdom; 4 MRC Laboratory for Molecular Cell Biology, University College London, Gower Street, London WC1E 6BT, United Kingdom; 5 UCL Cancer Institute, University College London, 72 Huntley Street, London WC1E 6DD, United Kingdom

**Keywords:** PNS regeneration, Schwann cell, dedifferentiation, Eph signaling, TGFb signaling, plasticity

## Abstract

Schwann cell dedifferentiation from a myelinating to a progenitor-like cell underlies the remarkable ability of peripheral nerves to regenerate following injury. However, the molecular identity of the differentiated and dedifferentiated states *in vivo* has been elusive. Here, we profiled Schwann cells acutely purified from intact nerves and from the wound and distal regions of severed nerves. Our analysis reveals novel facets of the dedifferentiation response, including acquisition of mesenchymal traits and a Myc module. Furthermore, wound and distal dedifferentiated Schwann cells constitute different populations, with wound cells displaying increased mesenchymal character induced by localized TGFβ signaling. TGFβ promotes invasion and crosstalks with Eph signaling via N-cadherin to drive collective migration of the Schwann cells across the wound. Consistently, *Tgfbr2* deletion in Schwann cells resulted in misdirected and delayed reinnervation. Thus, the wound microenvironment is a key determinant of Schwann cell identity, and it promotes nerve repair through integration of multiple concerted signals.

**Video Abstract:**

## Highlights

• Wound Schwann cells have a distinct dedifferentiation program
• TGFβ signaling reprograms wound Schwann cells to invasive mesenchymal-like cells
• TGFβ crosstalks with Eph signaling to drive collective Schwann cell migration
• Loss of TGFβ signaling in Schwann cells delays nerve regeneration

## Teaser

Clements et al. show that the nerve-injury-induced Schwann cell dedifferentiation program is modulated by the wound microenvironment through TGFβ. TGFβ promotes regeneration by reprogramming Schwann cells to invasive, mesenchymal-like cells and crosstalking with Eph signaling to drive collective migration.

## Introduction

The adult peripheral nervous system (PNS) retains significant regenerative potential, enabling the repair of even severe injuries such as full transection of the nerve trunk (Chen et al., 2007). Following a lesion to the nerve, the portion of the axons distal to the injury site degenerates. However, unlike central nervous system axons, damaged peripheral axons are able to regrow and reinnervate their targets (Chen et al., 2007). This process is underpinned by the remarkable plasticity of the PNS glia, the Schwann cells (SCs) (Jessen et al., 2015, Kim et al., 2013a). In intact nerves, SCs are present in two differentiated states, either myelinating large-caliber axons (myelinating SCs) or ensheathing groups of small-caliber axons in Remak bundles (nonmyelinating SCs). In contrast, following injury, differentiated SCs reprogram to a progenitor-like cell. Dedifferentiated SCs switch off the myelination program and acquire an array of new phenotypes, which coordinately support nerve repair. These phenotypes include 1) secretion of neurotrophic factors to promote axonal survival, 2) clearance of myelin debris and expression of axonal guidance and adhesive cues to generate a favorable environment for axonal regrowth, 3) initiation of an inflammatory response to promote wound healing, and 4) proliferation to replace lost cells (Jessen et al., 2015).

Furthermore, upon transection injuries that generate a gap in the nerve, dedifferentiated SCs guide regrowing axons through the nerve bridge, a piece of connective tissue that forms across the gap to rejoin the severed stumps (McDonald et al., 2006). SCs from both stumps invade the bridge as multicellular cords and migrate directionally, eventually rejoining. This generates continuous cellular conduits across the wound and along which axons regrow to cross the gap and reinnervate the distal stump (Jessen et al., 2015, Parrinello et al., 2010).

At the molecular level, cord migration is driven by Eph/ephrin-mediated interactions between SCs and wound fibroblasts. Activation of EphB2 receptors on the SCs by ephrin-B ligands on fibroblasts induces SC sorting into cords through the relocalization of N-cadherin to cell-cell contacts in a Sox2-dependent manner (Parrinello et al., 2010).

Seminal work over the past few decades has uncovered key transcription factors (e.g., c-*jun*, sox2), epigenetic changes, and signaling pathways (e.g., ERK, Notch) that control SC reprogramming (Arthur-Farraj et al., 2012, Hung et al., 2015, Jessen et al., 2015, Le et al., 2005, Napoli et al., 2012, Woodhoo et al., 2009). However, a comprehensive molecular characterization of myelinating and dedifferentiated SCs *in vivo* is still lacking. Furthermore, despite the specialized function of bridge SCs, it remains unclear whether the reprogrammed state is homogeneous throughout the injured nerve or whether regional differences in SC state may exist, particularly within the bridge.

This gap in our knowledge is largely due to the technical limitations of purifying sufficient numbers of SCs for standard downstream molecular analysis. Consistent with this, previous investigation of the transcriptional changes associated with nerve regeneration have been limited to analysis of whole nerves before and after injury (Arthur-Farraj et al., 2012, Jiang et al., 2014, Kubo et al., 2002, Le et al., 2005). However, interpretation of this type of analysis is confounded by the cellular heterogeneity of nerve tissue, the pronounced inflammatory response that ensues injury, and the difference in SC content between intact and injured nerves (Cattin et al., 2015, Jessen et al., 2015). Furthermore, whole-tissue approaches are not amenable to the analysis of bridge SCs due to the extensive influx of stromal and inflammatory cells into the nerve bridge. Nonetheless, deciphering the SC transcriptome and its modulation by the microenvironment is a prerequisite to the understanding of PNS regeneration and, more generally, of the molecular basis of somatic cell plasticity.

In this study, we developed a fluorescence-activated cell sorting (FACS)-based approach to prospectively purify SCs from mouse sciatic nerves. By combining this approach with RNA amplification protocols, we carried out transcriptional profiling of *in vivo* SCs from intact nerves and from bridge and distal stumps of transected nerves. Our analysis reveals novel phenotypes associated with SC reprogramming. Importantly, we also find that bridge SCs are a distinct subpopulation of progenitor-like SCs of increased mesenchymal character. We show that a localized increase in TGFβ signaling in the bridge underlies this change in order to promote SC invasion into the wound. Surprisingly, our studies also reveal an unprecedented crosstalk between TGFβ and EphB2, which we find is effected by transcriptional regulation of N-cadherin downstream of TGFβ.

Together, our findings indicate that extracellular cues from the wound microenvironment intersect with cell-intrinsic dedifferentiation mechanisms to reprogram the SC to an invasive, mesenchymal-like cell. They further identify TGFβ as a key mediator of peripheral nerve regeneration after transection and Eph/ephrin signaling as a novel TGFβ effector, with important implications for the EMT, wound healing, and cancer.

## Results

### Purification of Differentiated and Progenitor-like SCs

**Figure 1:**
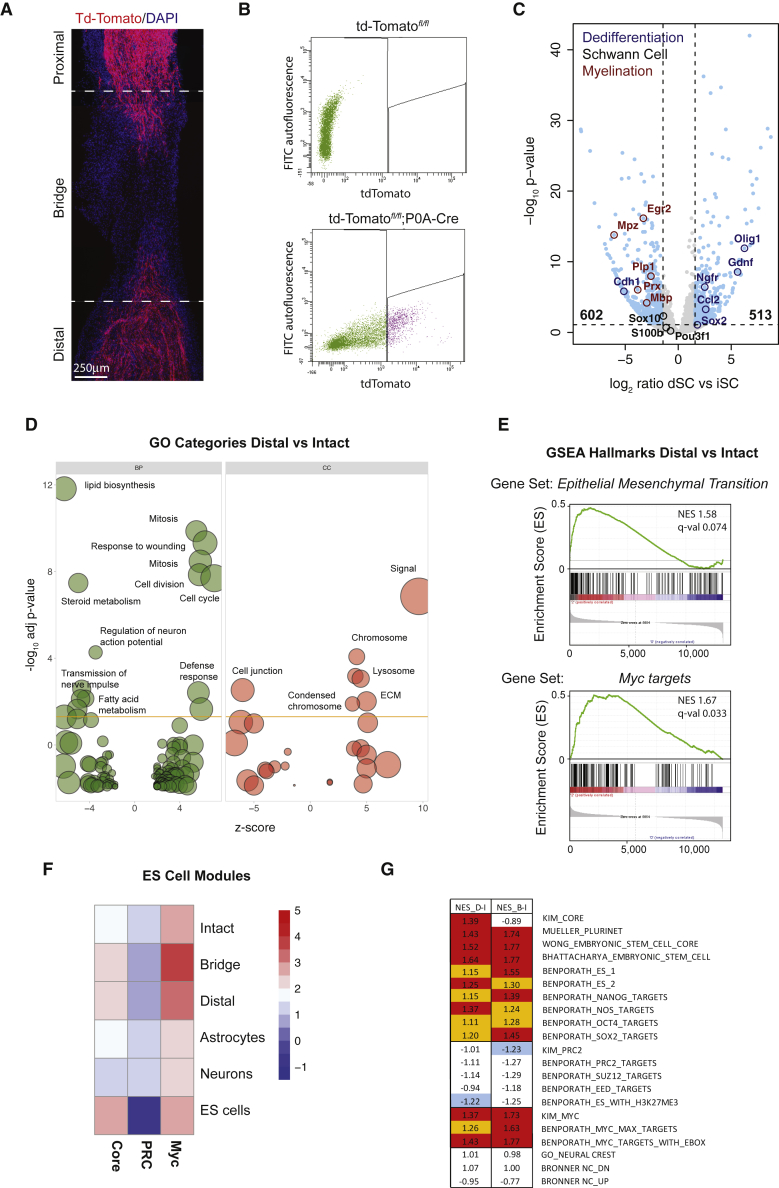
RNA Sequencing of Differentiated and Progenitor-like SCs Identifies Novel Features of Dedifferentiation (A) Example of a regenerating sciatic nerve of *tdTom*^*SC*^ mice collected 6 days post-transection. A representative tiled fluorescent image of the nerve regions collected for FACS purification is shown. tdTomato^+^ SCs are in red, and DAPI-stained nuclei are in blue. Dotted lines demarcate the nerve bridge. (B) Representative FACS plots of the purification of tdTomato^+^ SCs from sciatic nerves of *tdTom*^*SC*^ mice (bottom). Control *P0A*-Cre^−^;*tdTom*^*fl/fl*^ cells (top) were used for gating. (C) RNA-seq differential gene expression analysis of SCs from pooled distal stump (dSCs) and intact nerve (iSCs). Genes regulated over 2.5× (adj. p < 0.05, light blue), myelination genes (red circles), SC markers (black circles), and dedifferentiation markers (dark blue circles) are highlighted. (D) Functional analysis of genes differentially regulated between dSCs and iSCs. Genes with dSC:iSC count ratios >2.5× (adj. p < 0.05) were selected. –log_10_ of the enrichment p values for selected GO categories (BP: biological process, CC: cellular components) are plotted relative to *Z* scores of average dSC:iSC count ratios in each category. Circle size denotes the number of regulated genes. (E) GSEA enrichment plots of dSCs compared to iSCs for the “epithelial mesenchymal transition” and “Myc targets V1” MSigDB hallmarks. (F) Average log_2_ FPKM expression of the ES core, PRC, and Myc modules from (Kim et al., 2010) for iSCs, pooled SCs isolated from the nerve bridge (bSCs), and dSCs, as well as differentiated cells of neural origin (Astrocytes, Neurons) and pluripotent ES cells (ES cells) (Zhang et al., 2014, Marks et al., 2012). FPKM have been median centered to allow comparison across datasets. (G) GSEA enrichment analysis of dSCs compared to iSCs (D–I) and bSCs compared to iSCs (B–I) for MSigDB gene sets related to pluripotency (lines 2–10), regulation by the Prc2 complex (11–15), regulation by Myc (16-18), three ES modules (lines 1, 11, and 16), and markers of neural crest identity (19–21). Numbers represent normalized enrichment score and colors represent the FDR q values ≤ 0.1 (red) or ≤ 0.25 (orange/blue). See also Figure S1.

To isolate SCs from nerve tissue, we generated Schwann-cell-specific tdTomato reporter mice (hereafter *tdTom*^*SC*^) by crossing *P0A*-Cre animals, which express Cre recombinase under the control of the neural crest-specific Myelin Protein Zero (*Mpz*) promoter, to *Rosa26*^*tdTomato*^ reporter strains (Giovannini et al., 2000, Madisen et al., 2010). Consistent with previous studies, analysis of *tdTom*^*SC*^ mice revealed strong tdTomato expression, unaffected by injury, in adult sciatic nerves (Figures 1A and S1A). Furthermore, coimmunostaining for the SC markers MBP and p75^NGFR^, which specifically label myelinating and nonmyelinating SCs, respectively, confirmed that tdTomato recombination was efficient and restricted to SCs (Giovannini et al., 2000, Zheng et al., 2008) (Figures S1A and S1B). Thus, tdTomato is a suitable marker for purification of differentiated and dedifferentiated SCs from their *in vivo* microenvironment using FACS.

To this end, the right sciatic nerve of *tdTom*^*SC*^ mice was transected and, 6 days later, the contralateral intact nerve and distal stump of the severed nerve were collected together with the nerve bridge to determine potential effects of the wound microenvironment. This time point was specifically chosen because it coincides with complete SC dedifferentiation and extensive, but not complete, invasion into the bridge (Figure S2B) (Chen et al., 2007, Parrinello et al., 2010). Nerve tissue was dissociated to single cells, and SCs were sorted from other tdTomato^−^ nerve cells based on tdTomato expression (Figure 1B). Single cell preparations from *P0A-Cre*^−^ nerves were used as reference for setting FACS gates. Acute immunostaining and Calcein labeling of FACS-sorted tdTomato cells confirmed that tdTomato^+^ cells were highly enriched for tdTomato expression, contained negligible contamination of tdTomato^−^ cells, expressed the pan-SC marker S100β, and were viable (Figures S1C and S1D). FACS analysis of cell suspensions from all three nerve regions indicated that the vast majority of the tdTomato^+^ but almost none of the tdTomato^−^ cells were S100β^+^, further confirming the purity of sorted tdTomato cells (Figure S1E). Furthermore, qRT-PCR analysis indicated that tdTomato^+^ cells from intact and transected nerves were enriched for the appropriate SC differentiation (*Mpz*, *Plp*, *Pmp22*) and dedifferentiation (*p75*^*NGFR*^, *Sox2*, *c-Jun*) markers, respectively (Figure S1F). Therefore, our approach enables efficient purification of SCs from intact and severed nerve tissue.

We next characterized the *in vivo* SC transcriptome by high-thoughput sequencing of cDNA (RNA-seq). Libraries were prepared from four biological replicates per nerve region using a Smart-seq2 based protocol, generating an average of ∼33 × 10 reads per sample and leading to quantification of ∼15,000 genes (Picelli et al., 2014). Comparable transcriptome coverage was obtained from higher-RNA-input libraries derived from *in vitro* material, indicating that low RNA inputs did not compromise the sensitivity of our analysis (Table S1). Moreover, reproducibility of transcript level quantification between biological repeats was high, and PCA analysis separated the samples into three clearly distinct groups, which corresponded to nerve region of origin (Figures S1G and S1H).

Having confirmed the quality and reproducibility of the RNA-seq data, we first examined the transcriptional changes that accompany dedifferentiation by comparing the transcriptomes of SCs isolated from intact nerve (iSCs) and distal stump (dSCs) (Tables S2 and S3). Importantly, analysis of RNA spike-in controls was not supportive of a global relative increase in RNA polymerase II (RNAPII) transcription levels upon dedifferentiation (Figure S1I). Instead, differential expression analysis indicated that a relatively small proportion of the SC transcriptome changed significantly (1,692 genes; 1,115 with >2.5-fold change). This entailed similar numbers of up- and downregulated genes that included well-established markers for both populations, further corroborating the validity of our method (Figure 1C). Gene ontology (GO) and gene set enrichment analysis (GSEA) revealed several distinct biological features between differentiated and progenitor-like SCs. As expected, the transcriptome of dSCs was enriched for genes involved in cell cycle, immune recruitment, and phagocytosis, whereas GO terms associated with myelination and nerve conduction were significantly downregulated (Chen et al., 2007, Jessen et al., 2015) (Figure 1D). Moreover, our analysis revealed a switch in how SCs communicate with the surrounding nerve tissue, in that iSCs showed increased levels of cell-cell communication genes (cell junction), whereas dSCs favored expression of genes mediating communication with the extracellular matrix (extracellular signal and extracellular matrix). Notably, signatures of epithelial-to-mesenchymal transition (EMT) and activated Myc were among the most highly enriched GSEA gene sets in dSCs (Figure 1E).

The EMT has been linked to both somatic cell dedifferentiation to cancer stem cells and somatic cell reprogramming to induced pluripotent cells (iPS) (Ye and Weinberg, 2015). The transcription factor Myc is a crucial regulator of ES pluripotency and one of the original Yamanaka reprogramming factors (Takahashi and Yamanaka, 2016). Therefore, these signatures support the notion that dedifferentiation is a reprogramming event that converts SCs to progenitor-like cells (Arthur-Farraj et al., 2012). To investigate this further, we analyzed expression of the three transcriptional programs that define ES cell pluripotency (Kim et al., 2010). These comprise targets of core pluripotency factors (i.e., Sox2, Nanog, Oct4, Core module), polycomb-related factors, which repress lineage-specific genes (PRC module), and Myc target genes (Myc module). Publicly available RNA-seq datasets of ES cells and differentiated CNS cells (astrocytes and neurons) served as reference transcriptomes (Marks et al., 2012, Zhang et al., 2014) (Figure 1F). Remarkably, we found that dSCs activate Core and Myc modules while repressing the PRC module, a behavior reminiscent of ES cells (Kim et al., 2010). In contrast, module activity patterns of iSCs were similar to those of differentiated cells. Consistent with these results, GSEA analysis indicated that signatures of ES cells were highly enriched in dSCs (Figure 1G) (Ben-Porath et al., 2008, Bhattacharya et al., 2004, Kim et al., 2010, Müller et al., 2008, Wong et al., 2008), whereas signatures of the neural crest, from which the SCs derive, were not (Simões-Costa et al., 2014). These results suggest that, although lineage characteristics are retained after injury, dedifferentiation is not a reversal of the SC developmental program. Rather, injury reprograms SCs to a stem-like state.

### Distal and Bridge SCs have Distinct Molecular Features

**Figure 2:**
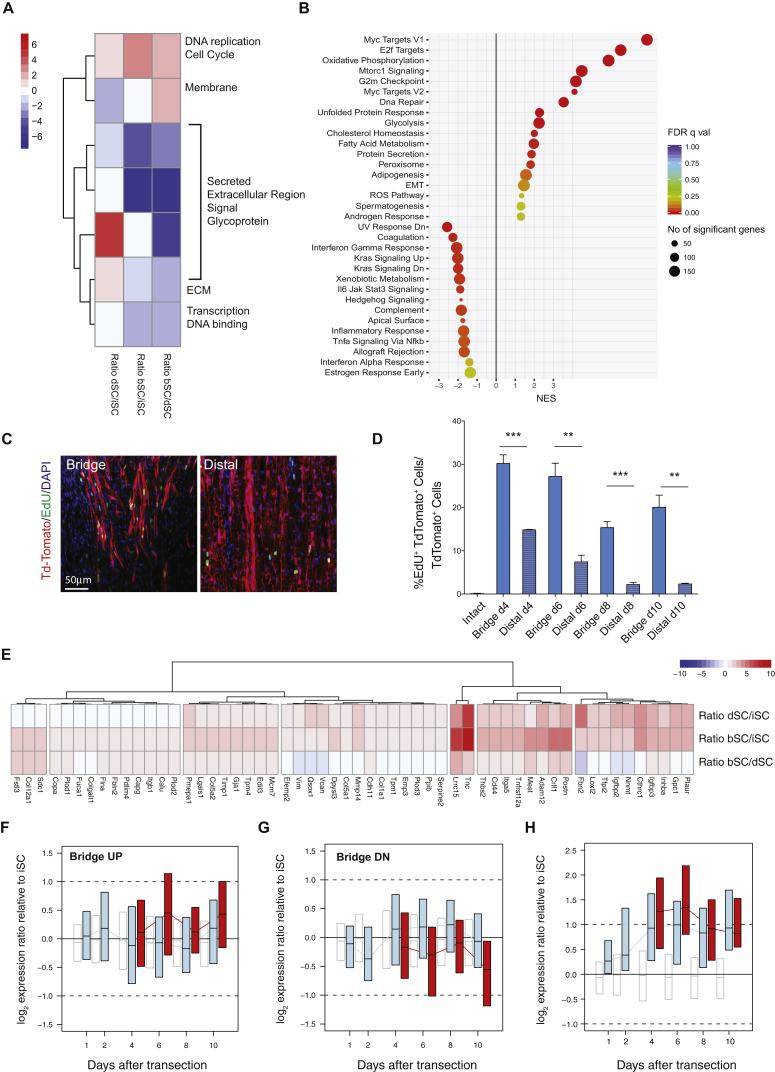
Bridge and Distal SCs Have Distinct Molecular Signatures (A) Seven k-means clusters of dSC:iSC, bSC:iSC and bSC:dSC expression ratios. Colors represent the mean of log_2_ expression ratios in each cluster and condition. Selected functional categories enriched in each cluster are shown. Cluster numbers from top to bottom: 2,6,3,4,7,1,5, as in Table S4. (B) GSEA enrichment analysis of preranked bSC:dSC ratios for MSigDB hallmarks. Gene sets with FDR q values <0.25 are plotted relative to normalized enrichment scores (NES). Categories with negative or positive NES are down- or upregulated, respectively, in bSCs. Circle size denotes the number of enriched genes in each category and circle colors represent FDR q values as indicated. (C) Representative EdU staining (green) of actively proliferating tdTomato^+^ SCs in the bridge and distal stump 6 days post-transection. (D) Quantification of EdU^+^/tdTomato^+^ SCs relative to total tdTomato^+^ SCs in the bridge and distal nerves at 4, 6, 8, and 10 days post-transection and in the intact nerve (mean ± SEM). n = 6 at day 6 and n = 3 at days 4, 8, and 10, ^∗∗^p < 0.01 and ^∗∗∗^p < 0.001, two-tailed paired Student’s t test. (E) Hierarchical clustering of log_2_ expression ratios for genes belonging to the “epithelial mesenchymal transition” MSigDB hallmark. Genes significantly upregulated (adj. p < 0.05) in bSCs compared to iSCs are shown. Rows are dSC:iSC, bSC:iSC, and bSC:dSC log_2_ ratios from top to bottom. (F–H) RNA-seq time course analysis of dSC and bSC gene expression changes after sciatic nerve transection. dSC:iSC (blue) or bSC:iSC (red) expression ratios are plotted relative to time after injury for the genes (F) upregulated in bSCs relative to dSCs from figure S2A, (G) downregulated in bSCs relative to dSCs from S2A, and (H) belonging to the EMT signature from (E). Boxes denote the interquartile range, and black strikes denote the median of ratios in the list. Open boxes represent dSC:iSC ratios for all genes as a reference. Data from cells isolated from single nerves are shown; n = 3–4 per time point. See also Figure S2.

We next compared the transcriptomes of dSCs and bridge SCs (bSCs) (Tables S2 and S3). Interestingly, the number of differentially expressed genes between bSCs and dSCs, despite both of these being in a dedifferentiated state, was comparable to the number detected between iSCs and dSCs (2,021 genes; 1,169 with >2.5-fold change), with an overall bias toward downregulation in the bridge (Figure S2A). We generated seven gene clusters by K-means clustering of expression ratios that were further examined by GO analysis and combined with GSEA analysis of the entire dataset (Figures 2A and 2B; Table S4). Our analysis revealed a downregulation in genes essential to the repair program in bSCs, such as genes involved in inflammation, immune signaling, and ECM production, and a decrease in transcriptional activity. In contrast, genes involved in cell division, growth, and metabolism were strongly upregulated, suggesting that bridge cells might have an increased proliferative capacity. To test this more directly, we measured cell proliferation *in vivo* by pulsing *tdTom*^*SC*^ mice with EdU for 4 hr from 4 to 10 days post-transection, a timeline encompassing all stages of SC migration into the bridge (Figure S2B). Consistent with the RNA-seq data, more EdU^+^ SCs were detected in the bridge than in distal stump at all time points (Figures 2C and 2D), indicating that bSCs are more proliferative than their distal counterparts.

GSEA analysis indicated that the EMT and active Myc signatures detected upon transition from iSCs to dSCs were further enriched in bSCs, indicative of a potential change in differentiation state (Figure 2B). Indeed, closer examination of the bridge EMT signature revealed that bSCs had a more pronounced mesenchymal signature than did dSCs, with many EMT genes being more highly expressed in bSCs than in dSCs (Figure 2E). Of note, a similar decrease in *Cdh1* expression, a hallmark of the EMT, was detected in both dSCs and bSCs (dSCs/iSCs: −5.13, p = 1.52e^−6^; bSCs/iSCs: −3.33, p = 2.5e^−3^). To further assess these differences and determine whether they are distinctive properties of bSCs throughout repair, we carried out time-course analysis of bSC and dSC transcriptomes by RNA-seq (Table S6). SCs were acutely FACS-sorted from the bridge and distal stumps of single transected or contralateral intact nerves. dSCs were analyzed at days 1, 2, 4, 6, 8, and 10; nerve-matched bSCs were analyzed from day 4, the earliest time point at which invasion into the bridge occurs, up to day 10 (Figure S2B). Importantly, this analysis revealed that the differences in gene expression between bSCs and dSCs observed at day 6 are established as soon as bSCs enter the bridge and are maintained over time (Figures 2F and 2G). Similarly, genes associated with repair (k-means clusters 1 and 7) were downregulated in bSCs at all time points (Figures S2C and S2D). In contrast, the increase in EMT genes was restricted to time points at which bSCs invade into the bridge, in that this increase began at day 4, peaked at day 6, and subsided at day 8, when invasion is largely complete (Figures 2H and S2B). Importantly, this enhanced EMT signature was never detected in dSCs, indicating that it is a bridge-specific phenotype (Figure 2H). Together, these results indicate that bridge SCs are a molecularly distinct subpopulation of dedifferentiated cells with reduced repair-promoting ability and with increased proliferation and differentiation toward the mesenchymal lineage.

### TGFβ Signaling Reprograms Bridge SCs to Invasive Mesenchymal-like Cells

**Figure 3:**
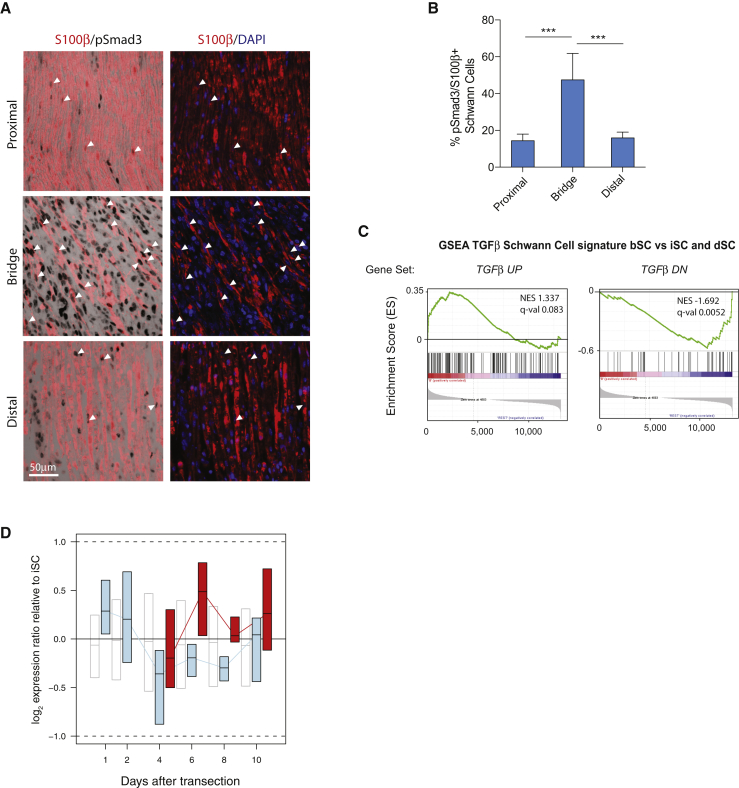
TGFβ Signaling Is Increased in the Nerve Bridge (A) p-Smad3 expression in the proximal (top), bridge (middle), and distal (bottom) nerve 6 days post-transection. Paraffin sections were stained for p-Smad3 (DAB), the SC marker S100β (red), and DAPI (blue). White arrowheads indicate p-Smad3 colocalization with SC nuclei. (B) Quantification of p-Smad3-expressing SCs in each region (mean ± SEM). n = 9, ^∗∗∗^p < 0.001, one-way ANOVA with Bonferroni correction. (C) GSEA enrichment plots of bSCs compared to dSCs and iSCs for the SC-specific transcriptional response to TGFβ (this study). TGFβ UP and TGFβ DN gene sets are up- or downregulated, respectively, by TGFβ. (D) RNA-seq time course analysis of dSC or bSC gene expression changes after sciatic nerve transection. dSC:iSC (distal:intact; blue) or bSC:iSC (bridge:intact; red) expression ratios are plotted relative to time after injury for the 30 TGFβ UP genes with the highest significant bSC:dSC ratios in the dataset in (C) (p < 0.05). Boxes denote the interquartile range, and black strikes denote the median of ratios in the list. Open boxes represent dSC:iSC expression ratios for all genes as a reference. Data from cells isolated from single nerves are shown; n = 3–4 per time point. See also Figure S3.

The ECM and cellular composition of the bridge microenvironment is markedly different from the rest of the injured nerve (Cattin et al., 2015, McDonald et al., 2006, Parrinello et al., 2010). We therefore hypothesized that wound-specific extracellular signals might underlie the distinct molecular profile of bSCs. TGFβ is a particularly good candidate for this because it is a master regulator of the EMT and wound healing and has been previously linked to PNS regeneration (D’Antonio et al., 2006, Lamouille et al., 2014; reviewed in Mirsky et al., 2008). We therefore examined TGFβ activity in the regenerating nerve using two approaches. First, we coimmunostained wild-type nerves 6 days after cut for phosphorylated Smad3 (p-Smad3), the canonical downstream effector of the TGFβ pathway (Shi and Massagué, 2003), and also S100β, which labels all SCs in the injured nerve (Figure S1E). Consistent with previous reports of increased TGFβ levels upon injury, p-smad3^+^/S100β^+^ cells could be detected throughout the cut nerve, but their number strongly increased in the bridge, indicating that TGFβ signaling activity is maximal in bSCs (Figures 3A and 3B) (Mirsky et al., 2008). Similar results were obtained in *tdTom*^*SC*^ nerves (Figure S3A). Second, we interrogated the *in vivo* SC transcriptome for signatures of active TGFβ signaling. To generate Schwann-cell-specific TGFβ signatures, we performed RNA-seq of *in vitro* rat SCs that were cultured in the presence or absence of recombinant TGFβ for 16 hr. Transcripts differentially expressed in response to TGFβ treatment were used as signature gene sets for GSEA analysis of the *in vivo* datasets (Table S5). As shown in Figure 3C, we found that the Schwann-cell-specific TGFβ signatures were enriched significantly in bSCs, but not in iSCs or dSCs. To further assess TGFβ activity throughout the formation and invasion of bSC cords, we next examined TGFβ signature genes in the RNA-seq time course data. We found that TGFβ target gene expression is increased in bSCs relative to dSCs at all time points, confirming that TGFβ signaling is highest in invading bSCs within regenerating cut nerves (Figure 3D). Consistent with these findings, TGFβ mRNA levels were highly enriched in the bridge microenvironment; this was judged by qRT-PCR analysis of acutely FACS-sorted tdTomato^−^ bridge cells compared to tdTomato^+^ SCs (Figure S3B). Furthermore, the bridge contained large numbers of p-Smad3^+^/S100β^−^ cells—many of which were fibroblasts and endothelial cells, the most abundant cell types in this region—and fibroblasts expressed higher levels of TGFβ than SCs *in vitro* (Figures S3C–S3E) (Cattin et al., 2015, Parrinello et al., 2010).

To understand the functional role of TGFβ signaling in the bridge, genes that were commonly upregulated in both bSCs and TGFβ-treated rat SCs were compared to GSEA Hallmark gene sets. This identified the EMT signature as the most significantly enriched gene set (p = 5.86e^−15^), indicating that TGFβ may be primarily involved in driving the increased mesenchymal transition of bSCs. In agreement with this, qRT-PCR analysis confirmed that many bSC-specific EMT genes were TGFβ targets (Figure S4A).

Next, we assessed the effects of loss of TGFβ function in bSCs. We ablated TGFβ receptor (TGFR) activity specifically in SCs by crossing the *P0A-Cre* strain to mice carrying conditional *Tgfbr2* alleles, which encode for the obligatory component of the TGFβ receptor complex TGFβ receptor 2 protein (hereafter referred to as *Tgfbr2*^*ΔSC*^) (Levéen et al., 2002). Genomic recombination of the *Tgfbr2*-floxed alleles in the adult sciatic nerves of *Tgfbr2*^*ΔSC*^ mice was confirmed by PCR analysis (Figure S4B). To assess recombination efficiency and functional inactivation of the pathway, *Tgfbr2*^*fl/fl*^ and *Tgfbr2*^*ΔSC*^ nerves were immunostained for p-smad3 and S100β, as in Figure 3. This showed efficient, but variable, SC-specific loss of TGFR activity (60% to 80% of all SCs), consistent with previous studies (Figures S4C and S4D) (Giovannini et al., 2000, Zheng et al., 2008). Importantly, ultrastructural analysis by electron microscopy of intact *Tgfr2*^*ΔSC*^ adult sciatic nerves revealed that they were similar to controls, indicating that this model can be used to study PNS regeneration in the absence of confounding developmental defects (Figures S4E–S4G).

**Figure 4:**
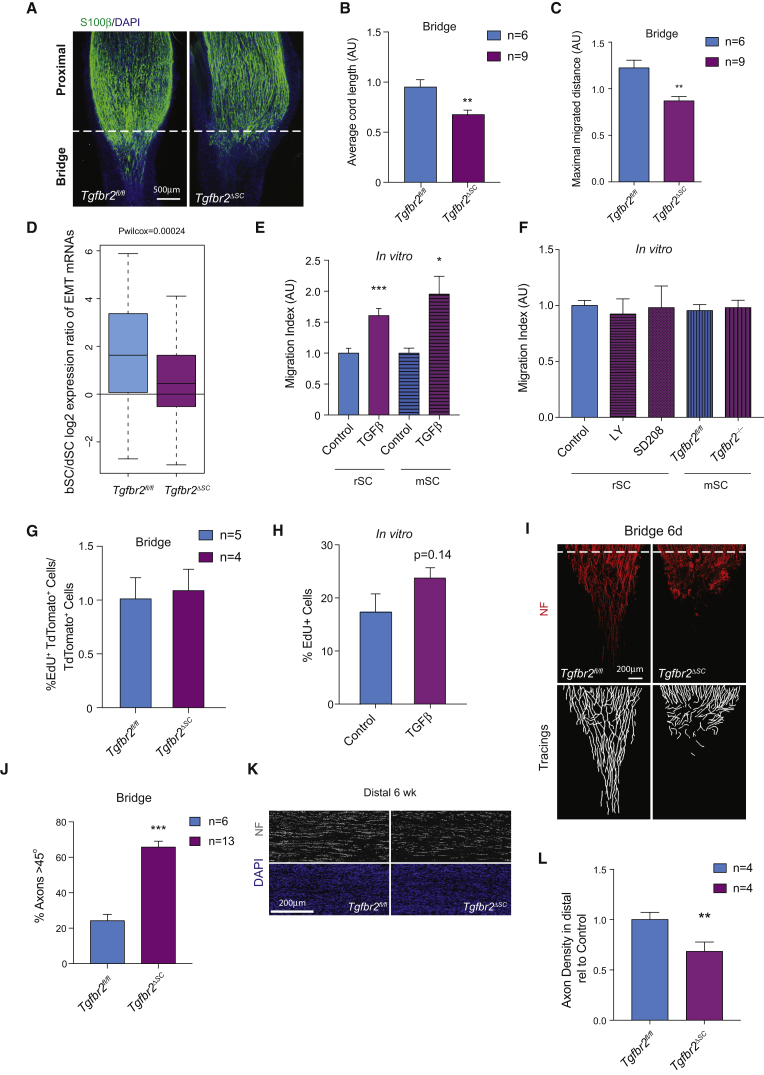
TGFβ Drives SC Invasion into the Bridge (A) Representative immunofluorescence staining for the SC marker S100β (green) in longitudinal sections of regenerating proximal stumps of *Tgfbr2*^*ΔSC*^ mice and P0A-Cre^−^ control littermates *Tgfbr2*^*fl/fl*^ 6 days post-transection. Nuclei are stained with DAPI. Dashed lines mark the border between the proximal stump and the nerve bridge. (B) Average length of the SC cords from the *Tgfbr2*^*ΔSC*^ and *Tgfbr2*^*fl/fl*^ cut nerves from (A) (mean ± SEM). n = 6 for *Tgfbr2*^*fl/fl*^, n = 9 for *Tgfbr2*^*ΔSC*^, ^∗∗^p < 0.01, two-tailed paired Student’s t test. (C) Maximal migrated distance of the SC cords from *Tgfbr2*^*ΔSC*^ and *Tgfbr2*^*fl/fl*^ cut nerves from (A) (mean ± SEM). n = 6 for *Tgfbr2*^*fl/fl*^, n = 9 for *Tgfbr2*^*ΔSC*^, ^∗∗^p < 0.01, two-tailed paired Student’s t test. (D) qRT-PCR analysis of the bridge-specific mesenchymal gene signature in bSCs and dSCs FACS-sorted from single nerves of *tdTom;Tgfbr2*^*fl/fl*^ and *tdTom;Tgfbr2*^*ΔSC*^ mice. Boxplots represent log_2_ bSC:dSC expression ratios of 24 bridge-specific EMT genes. Pooled data for *Tgfrb2*^*fl/fl*^ (blue bar, n = 6) and *Tgfbr2*^*ΔSC*^ (purple bar, n = 5) mice are shown; p_Wilcoxon_ = 0.00024. The whiskers extend to the most extreme data point, which is no more than 1.5× the interquartile range from the box. (E) SC invasion through fibronectin-coated Boyden chambers in the absence or presence of TGFβ. Rat SCs (rSC, solid bars) and mouse SCs (mSC, hatched bars) were treated with vehicle (blue bar) or TGFβ (purple bar, 10 ng/mL) (mean ± SEM). n = 3, ^∗^p < 0.05, ^∗∗∗^p < 0.001, one-way ANOVA. (F) SC invasion through fibronectin-coated Boyden chambers upon inhibition of TGFR signaling. Rat SCs (rSC) were left untreated or treated with TGFR inhibitors LY2157299 (LY; purple hatched bar) or SD208 (SD; purple spotted bar) for 24 hr prior to the assay. Mouse SCs (mSC) were either wild-type (*Tgfbr2*^*fl/fl*^, blue vertical lines) or knockout (*Tgfbr2*^*−/−*^; purple vertical lines) (mean ± SEM). n = 3. (G) EdU incorporation in the bridges of *tdTom;Tgfbr2*^*fl/fl*^ (blue bar, n = 5) and *tdTom;Tgfbr2*^*ΔSC*^ (purple bar, n = 4) mice 6 days post-transection. EdU incorporation in *tdTom;Tgfbr2*^*ΔSC*^ is normalized to controls (mean ± SEM). (H) EdU incorporation in rat SCs treated with vehicle (Control, blue bar) or TGFβ (TGFβ, purple bar) for 16 hr *in vitro* (mean ± SEM). n = 3, p = 0.14, two-tailed paired Student’s t test. (I) Representative immunofluorescence staining for the axonal marker neurofilament (red) in longitudinal sections of regenerating proximal stumps of *Tgfbr2*^*ΔSC*^ mice and P0A-Cre^−^ control littermates (*Tgfbr2*^*fl/fl*^) 6 days post-transection (Top). Dashed lines mark the border between the proximal stump and the bridge. The bottom panels show axonal tracings of images from the top panels obtained in NeuronJ. (J) Angles of axonal regrowth relative to the long axis of the nerves. Means ± SEM of the percentage of axons at angles >45° per group are shown. n = 6 for *Tgfbr2*^*fl/fl*^ and n = 13 for *Tgfbr2*^*ΔSC*^, ^∗∗∗^p < 0.001, two-tailed paired Student’s t test. (K) Analysis of distal stump reinnervation 6 weeks post-transection in *Tgfbr2*^*fl/fl*^ and *Tgfbr2*^*ΔSC*^ mice. Representative images of neurofilament staining in distal nerves are shown. (L) Quantification of neurofilament staining in the distal nerves of *Tgfbr2*^*fl/fl*^ and *Tgfbr2*^*ΔSC*^ mice shown in (K). Axon density was measured by fluorescence quantification in ImageJ. Data are normalized to *Tgfbr2*^*fl/fl*^ controls (blue bar) ± SEM n = 4 for each set, ^∗∗^p < 0.01, two-tailed paired Student’s t test. See also Figure S4.

Six days following transection, *Tgfbr2*^*ΔSC*^ and *P0A-Cre*^*−*^ *Tgfbr2*^*fl/fl*^ control nerve bridges were immunostained for S100β, and SC regrowth from the proximal stump was examined by quantifying the average SC cord length and maximal migrated distance into the bridge. This revealed a marked defect in bridge regeneration in *Tgfbr2*^*ΔSC*^ compared to controls (Figure 4A). In control nerves, numerous parallel SC cords invaded and migrated robustly into the bridge, as expected (McDonald et al., 2006, Parrinello et al., 2010). In contrast, in *Tgfbr2*^*ΔSC*^ mice, SC invasion was uneven and severely stunted, with cells extending much shorter distances from the proximal stump (Figures 4B and 4C). To determine whether this phenotype depended on loss of TGFβ-induced mesenchymal traits, we crossed *Tgfbr2*^*ΔSC*^ to *tdTomato* reporter mice, FACS-sorted bSCs from day-6 cut nerves of the resulting *tdTom*;*Tgfbr2*^*ΔSC*^ and littermate controls, and measured expression of bSCs-specific EMT genes by qRT-PCR. We found that the EMT signature was significantly downregulated in bSCs, but not in dSCs of *Tgfbr2*^*ΔSC*^ (Figures 4D, S4J, and S4K). In addition, we carried out Boyden chamber assays and found that TGFβ treatment significantly increases the ability of cultured rat and *Tgfbr2*^*fl/fl*^ mouse SCs to invade through fibronectin, a major component of the bridge ECM (Figure 4E) (Cattin et al., 2015). In contrast, although SCs themselves produce TGFβ (Figure S3E), neither pharmacological inhibition of TGFR in rat cells using two highly selective inhibitors (Akhurst and Hata, 2012) nor genetic deletion of *Tgfbr2* in mouse SCs that were freshly isolated from *Tgfbr2*^*fl/fl*^ mice affected invasion (Figures 4F, S4H, and S4I). Together, these results indicate that basal TGFR signaling is dispensable for sustaining the invasive ability of SCs. Rather, activation of TGBR by extrinsic TGFβ in the bridge enhances SC invasiveness.

TGFβ was shown to drive proliferation of cultured SCs in the presence of Neuregulin-1, a key SC mitogen (Parkinson et al., 2001). This raised the possibility that wound TGFβ might also be responsible for the increased proliferation of bSCs. We therefore measured EdU incorporation in bSCs of *tdTom*;*Tgfbr2*^*ΔSC*^ and control mice. As shown in Figure 4G, we found no significant difference in the number of EdU^+^/tdTomato^+^ bSCs, indicating that TGFβ does not regulate SC proliferation within the bridge. Consistent with this, TGFβ treatment of SCs cultured in serum, a condition that mimics the wound environment, did not significantly affect their proliferation (Figure 4H). We conclude that TGFβ induces a specialized mesenchymal transition in bSCs to increase their invasiveness, whereas bSCs proliferation is TGFβ independent.

### TGFβ Potentiates and Enables Eph-Dependent SC Sorting

While analyzing the regenerating nerve bridge of *Tgfr2*^*ΔSC*^, we noticed that SC cord migration into the bridge was highly disorganized. As regrowing axons comigrate with SCs from the proximal stump, we examined whether this aberrant SC migratory pattern also affected axons (Cattin et al., 2015, McDonald et al., 2006, Parrinello et al., 2010). Immunostaining for axonal markers revealed that whereas in control mice axonal regrowth into the bridge was directed toward the distal stump, regrowing *Tgfbr2*^*ΔSC*^ axons were disordered and misdirected. Indeed, quantification of the angles of axonal regrowth relative to the long axis of the nerve showed a marked increase in the percentage of axons that grew at >45° angles in *Tgfbr2*^*ΔSC*^ compared to controls (Figures 4I and 4J). The defect in SC and axonal migration into the bridge observed in *Tgfbr2*^*ΔSC*^ mice resulted in a significant decrease in reinnervation of the distal stump at 6 weeks post-transection (Figures 4K and 4L). However, no significant differences in the number or remyelination of distal axons were detected by electron microscopy at 3 months, suggesting that compensatory mechanisms enable long-term regeneration (data not shown).

**Figure 5:**
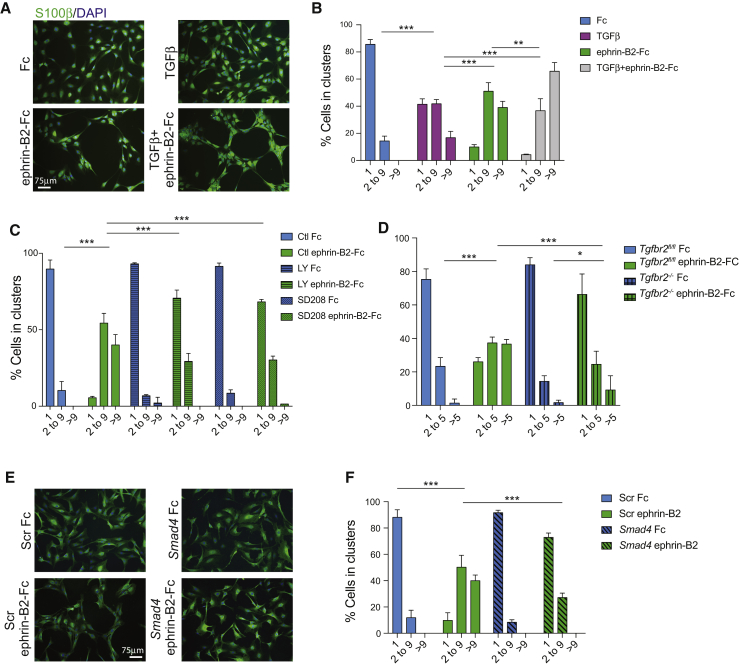
TGFβ Crosstalks with EphB2 to Modulate SC Sorting (A) Fluorescence images of rat SCs stained for S100β (green) plated on Fc control or ephrin-B2-Fc ligand in the presence or absence of TGFβ. (B) Quantification of cell sorting shown in (A) (mean ± SEM). n = 3, ^∗∗^p < 0.01; ^∗∗∗^p < 0.001, Fisher’s exact test. (C) Quantification of cell sorting as in (B) of rat SCs plated on Fc or ephrin-B2-Fc in the absence or presence of TGFR inhibitors LY2157299 (LY) or SD208 (SD). Cell sorting data is represented as mean ± SEM n = 3, ^∗∗∗^p < 0.001, Fisher’s exact test. (D) Quantification of cell sorting as in (B) for wild-type (*Tgfbr2*^*fl/fl*^) and knockout (*Tgfbr2*^*−/−*^) mouse SCs plated on Fc or ephrin-B2-Fc (mean ± SEM). n = 3, ^∗^p < 0.05; ^∗∗∗^p < 0.001, Fisher’s exact test. (E) Representative fluorescence images of rat SCs treated with Scr siRNA or siRNA against *Smad4* (*siSmad4*) and cultured on Fc or ephrin-B2-Fc recombinant proteins. (F) Quantification of cell sorting from (E) (mean ± SEM). n = 3, ^∗∗∗^p < 0.001, Fisher’s exact test. See also Figure S5.

This phenotype is remarkably similar to the regeneration defect of EphB2 knockout mice, raising the interesting possibility that the two pathways might interact *in vivo* (Parrinello et al., 2010). We previously reported that treatment of cultured SCs with recombinant ephrin-B2 ligands recapitulates their *in vivo* cell sorting behavior in response to bridge fibroblasts, resulting in the grouping of SCs into defined clusters (Parrinello et al., 2010). We therefore used this assay to examine effects of TGFβ on EphB2-mediated SC sorting. SCs were cultured on control Fc or ephrin-B2-Fc proteins in the presence or absence of TGFβ for 16 hr, and clustering was scored following immunostaining for S100β. Surprisingly, we found that TGFβ induced mild clustering on its own and significantly enhanced cell sorting in response to ephrin-B2, leading to larger and more compact clusters (Figures 5A and 5B). Furthermore, we examined the effects of loss of TGFβ activity on ephrin-B2-induced cell sorting in rat SCs cultured in the presence or absence of TGFR inhibitors and in freshly isolated *Tgfbr2*^*−/−*^ and *Tgfbr2*^*fl/fl*^ mouse cells. In both experiments, cell sorting was impaired (Figures 5C and 5D), indicating that EphB2 is strictly dependent on TGFβ signaling for function. These effects were mediated by Smad-dependent transcription downstream of TGFR because siRNA to the Co-Smad *Smad4*, which is required for nuclear translocation and transcriptional activity of p-Smads and which specifically inactivated TGFβ signaling in our system (Figure S5A), also severely disrupted SC clustering in response to ephrin-B2 (Figures 5E and 5F) (Shi and Massagué, 2003). Efficiency and specificity of the knockdown were confirmed by western blot analysis and parallel cell-sorting experiments, respectively, using a second independent siRNA oligo (Figures S5B, S5C, 6B, and 6I). Thus, TGFβ potentiates EphB2-dependent cell sorting, and its absence abolishes EphB2 function.

**Figure 6:**
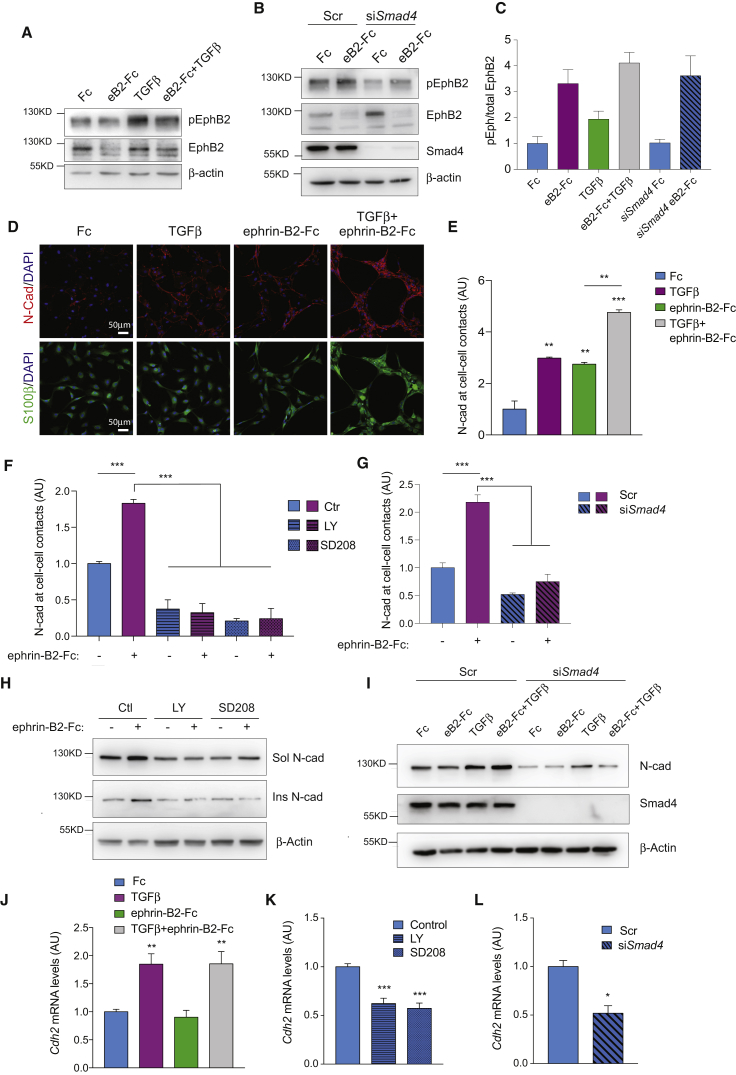
The TGFβ/EphB2 Crosstalk Is Mediated by N-Cadherin (A) Western blot analysis of p-EphB2 and total EphB2 levels in rat SCs seeded on Fc control or ephrin-B2-Fc ligands (eB2-Fc) in the presence of TGFβ or with ephrin-B2-Fc and TGFβ combined for 16 hr. β-actin is used as a loading control. (B) Western blot analysis of p-EphB2 and total EphB2 levels in rat SCs treated with Scr siRNA or siRNA against *Smad4* (si*Smad4*) and seeded on Fc control or ephrin-B2-Fc (eB2-Fc) ligands for 16 hr. Smad4 indicates knock down efficiency and β-actin loading. (C) Densitometric quantification of western blots shown in (A) and (B) obtained in FiJi (mean ± SEM), n=3. (D) Representative confocal images of rat SCs treated with the indicated ligands for 16 hr and stained for S100β (green) and N-cadherin (red). (E) Quantification of N-cadherin fluorescence at cell-cell junctions in the conditions shown in (D). Bar graphs show pixel intensities normalized by cell number (mean ± SEM). n = 3, ^∗∗^p < 0.01; ^∗∗∗^p < 0.001, two-tailed paired Student’s t test. (F) Quantification of N-cadherin fluorescence at cell-cell junctions in SCs treated with Fc (−) or ephrin-B2-Fc (+) in the absence (Ctr) or presence of LY2157299 (LY) and SD208 (SD) TGFR inhibitors. Means ± SEM are shown; n = 3, ^∗∗∗^p < 0.001, one-way ANOVA. (G) Quantification of N-cadherin fluorescence at cell-cell junctions in Scr and *Smad4* knockdown SCs treated with Fc (−) or ephrin-B2-Fc (+). Means ± SEM are shown; n = 3, ^∗∗∗^p < 0. 001, one-way ANOVA. (H) Western blot analysis of actin-bound and soluble N-cadherin in SCs cultured on Fc (−) or ephrin-B2-Fc (+) in the absence (Ctl) or presence of LY2157299 (LY) and SD208 (SD) TGFR inhibitors. β-actin is shown for loading. (I) Western blot analysis of total N-cadherin in Scr and *Smad4*-knockdown SCs plated on Fc and ephrin-B2-Fc in the presence or absence of TGFβ (TGFβ; eB2-TGFβ). Smad4 indicates knockdown efficiency and β-actin loading. (J) Quantitative RT-PCR analysis of *Cdh2* levels in SCs treated with Fc, TGFβ, ephrin-B2-Fc, and TGFβ and ephrin-B2-Fc combined, as indicated. Fold changes relative to Fc-treated controls are shown (mean ± SEM). n = 6, ^∗∗^p < 0.01, two-tailed paired Student’s t test. (K) qRT-PCR analysis of *Cdh2* levels in SCs cultured in the absence (Control) or presence of LY2157299 (LY) and SD208 TGFR inhibitors, as in (J). Means ± SEM are shown. n = 8, ^∗∗∗^p < 0.001, two-tailed paired Student’s t test. (L) Quantitative RT-PCR analysis of *Cdh2* levels in SCs treated with Scr siRNA or siRNA against *Smad4* (si*Smad4*). Means ± SEM are shown. n = 4, ^∗^p < 0.05, two-tailed paired Student’s t test.

### N-Cadherin Mediates TGFβ/Eph Crosstalk

We next determined the mechanisms by which TGFβ modulates the EphB2 pathway. On activation by ephrin-B ligands, EphB2 becomes phosphorylated (p-EphB2) and induces cell sorting by post-translationally modifying and stabilizing the pluripotency factor Sox2, which, in turn, increases Schwann cell-cell adhesion via relocalization of N-cadherin to cell-cell contacts (Parrinello et al., 2010). We therefore asked whether TGFβ directly modulates any of these pathway components. Western blot analysis indicated that EphB2 phosphorylation was largely unaffected by either TGFβ treatment or *Smad4* knockdown (Figures 6A–6C). Of note, a small increase in phosphorylation levels was detected in TGFβ samples. However, it was not accompanied by EphB2 internalization, a hallmark of activation, indicating that it did not elicit significant signaling (Figures 6A and 6B). Similarly, whereas ephrin-B2 stimulation increased Sox2 levels as expected (Parrinello et al., 2010), TGFβ treatment did not (Figure S6). In contrast, analysis of N-cadherin in cell-sorting assays revealed marked regulation by TGFβ. TGFβ alone relocalized N-cadherin to Schwann cell-cell contacts to a similar extent as ephrin-B2 ligands alone, and the two ligands combined synergised to potentiate this effect (Figures 6D and 6E). Conversely, treatment with TGFR inhibitors or *Smad4* knockdown abrogated the ability of ephrin-B2 to redistribute N-cadherin (Figures 6F and 6G). This was paralleled by changes in N-cadherin protein levels; TGFβ increased N-cadherin levels, whereas *Smad4* knockdown and inhibitor treatment strongly reduced basal N-cadherin levels, depleting both soluble and actin-bound N-cadherin pools (Figures 6H and 6I).

N-cadherin is a key EMT gene and a well-established TGFβ target (Yang et al., 2015). To test whether TGFβ may regulate *Cdh2*, the N-cadherin encoding gene, at the transcriptional level, we performed qRT-PCR analysis. We found that whereas *Cdh2* mRNA levels were unaffected by treatment with ephrin-B2 on its own, as expected, exposure to TGFβ alone or with ephrin-B2 significantly increased *Cdh2* expression. Inhibition of TGFβ signaling had the opposite effect, reducing *Cdh2* mRNA levels by about 50% (Figures 6J–6L) (Parrinello et al., 2010).

**Figure 7:**
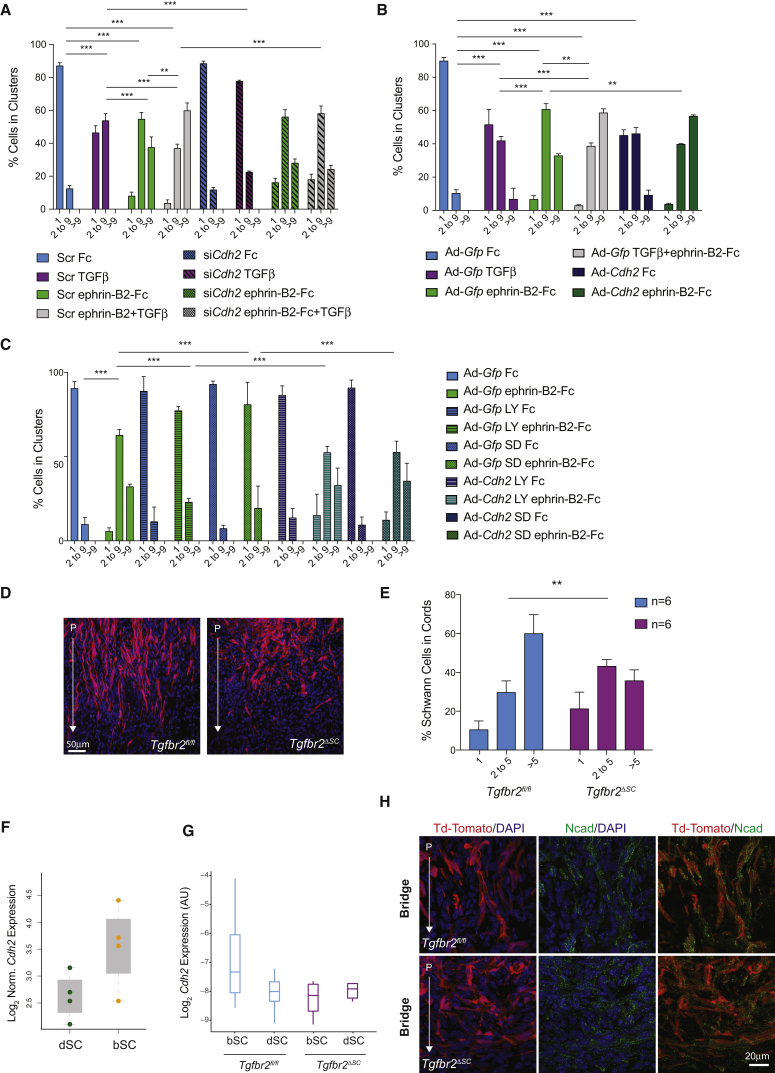
An Increase in N-Cadherin Levels Mediates TGFβ Effects on EphB2-Dependent Cell Sorting In Vitro and In Vivo (A) Quantification of cell sorting in Scr or partial *Cdh2* knockdown in rat SC cultures treated with Fc, TGFβ, ephrin-B2, and TGFβ and ephrin-B2 combined, as indicated. Cell sorting data is represented as mean ± SEM. n = 3, ^∗∗^p < 0.01; ^∗∗∗^p < 0.001, Fisher’s exact test. (B) Quantification of cell sorting in rat SCs transduced with adenoviruses encoding GFP (Ad-*Gfp*) or *Cdh2* (Ad-*Cdh2*) and treated with Fc, TGFβ, ephrin-B2-Fc, or TGFβ and ephrin-B2-Fc combined, as indicated (mean ± SEM). n = 3, ^∗∗^p < 0.01; ^∗∗∗^p < 0.001, Fisher’s exact test. (C) Quantification of cell sorting in rat SCs transduced with adenoviruses encoding GFP (Ad-*Gfp*) or *Cdh2* (Ad-*Cdh2*) and treated with Fc or ephrin-B2-Fc in the absence or presence of LY2157299 (LY) and SD208 (SD) inhibitors, as indicated (mean ± SEM). n = 3, ^∗∗∗^p < 0.001, Fisher’s exact test. (D) *In vivo* cell sorting in *tdTom;Tgfbr2*^*fl/fl*^ and *tdTom;Tgfbr2*^*ΔSC*^ in the bridge. Representative images of SC cords invading the bridge in *tdTom;Tgfbr2*^*fl/fl*^ and *tdTom;Tgfbr2*^*ΔSC*^ at 6 days post-transection. (E) Quantification of the *in vivo* cell sorting shown in panel D. Numbers of SCs in cords proximal to the migration front in *tdTom;Tgfbr2*^*fl/fl*^ and *tdTom;Tgfbr2*^*ΔSC*^ bridges are shown (mean ± SEM). n = 6 for each genotype, ^∗∗^p < 0.01, Fisher’s exact test. (F) Boxplot of RNA-seq FPKM expression scores for *Cdh2* in dSCs and bSCs. Colored dots represent single biological repeats. The whiskers extend to the most extreme data point, which is no more than 1.5× the interquartile range from the box. (G) qRT-PCR analysis of *Cdh2* levels in bSCs and dSCs from single nerves 6 days post-transection from *Tgfbr2*^*fl/fl*^ (blue bars, n = 4–5) and *Tgfbr2*^*ΔSC*^ mice (purple bars, n = 3–5). The whiskers extend to the most extreme data point, which is no more than 1.5× the interquartile range from the box. (H) Representative fluorescence images of N-cadherin protein levels in longitudinal sections of nerve bridges from *tdTom;Tgfbr2*^*fl/fl*^ (top) and *tdTom;Tgfbr2*^*ΔSC*^ (bottom) mice 6 days post-transection. P marks the proximal side and the arrow marks the direction of SC invasion. See also Figure S7.

To functionally assess whether transcriptional regulation of N-cadherin mediates the TGFβ/EphB2 crosstalk, we carried out gain- and loss-of-function cell sorting experiments. First, we knocked down *Cdh2* using a concentration of siRNA oligos sufficient to block its increase in response to TGFβ but insufficient to significantly alter *Cdh2* basal levels and thus inhibit basal ephrin-B2-induced clustering (Figure S7A). This partial *Cdh2* knockdown abolished clustering in TGFβ samples and reduced cell sorting to the levels of ephrin-B2-treated samples in cells treated with both ligands (Figure 7A). Then we ectopically increased *Cdh2* expression by adenoviral transduction to a level similar to TGFβ treatment (Ad-*Cdh2* cells, Figure S7B) and quantified cell sorting in the presence or absence of ephrin-B2 relative to GFP-transduced controls (Ad-*Gfp* cells). We found that untreated Ad-*Cdh2* cells phenocopied TGFβ-treated Ad-*Gfp* cultures and that treatment of Ad-*Cdh2* cells with ephrin-B2 ligands closely mimicked the synergistic effects of TGFβ and ephrin-B2 treatment combined (Figure 7B). Furthermore, increased *Cdh2* levels fully rescued ephrin-B2-induced cell sorting in the presence of TGFR inhibitors (Figure 7C). Together, these results indicate that the increase in *Cdh2* expression is both necessary and sufficient to potentiate cell sorting downstream of TGFβ.

Finally, we examined the relevance of these findings to TGFβ-mediated nerve regeneration *in vivo*. We first assessed effects of *Tgfbr2* loss on SC cords in the bridge. We found a decrease in the number of SCs in cords and an increase in single cells indicative of impaired EphB2-mediated cell sorting (Figures 7D and 7E). Then we analyzed N-cadherin expression *in vivo*. Consistent with a bridge-specific function, N-cadherin levels were 1.96-fold higher in bSCs than in dSCs in our RNA-seq dataset (Figure 7F). In addition, qRT-PCR analysis indicated that, although variable due to the low RNA input and the variable recombination efficiency seen in the *Tgfbr2*^*ΔSC*^ mice, *Cdh2* levels trended toward downregulation in *Tgfbr2*^*ΔSC*^ bSCs compared to *Tgfbr2*^*fl/fl*^ bSCs, but remained unchanged in dSCs (Figure 7G). At the protein level, immunofluorescence analysis indicated that whereas N-cadherin was highly enriched at Schwann cell-cell contacts in the bridges of control nerves, the protein appeared mislocalized and more diffuse in *Tgfbr2*^*ΔSC*^ nerves (Figure 7H). This difference was not due to axonal *Cdh2* expression; colocalization of neurofilament and N-cadherin proteins was minimal, and similar numbers of axonal fibers were seen in both genotypes (Figure S7C). Together, these observations confirm that N-cadherin is a TGFβ effector within regenerating nerves *in vivo*.

## Discussion

Since the groundbreaking discovery that differentiated somatic cells can be induced to become pluripotent by overexpression of defined transcription factors, the molecular basis of experimental reprogramming has been the focus of intense investigation (Merrell and Stanger, 2016, Takahashi and Yamanaka, 2016). However, increasing evidence suggests that, at least in some adult mammalian tissues, reprogramming can also occur naturally in response to injury (Merrell and Stanger, 2016). One of the most striking examples of physiological reprogramming is the dedifferentiation of SCs that occurs in peripheral nerve regeneration (Jessen et al., 2015, Kim et al., 2013a).

Here we developed a method to isolate pure populations of differentiated and dedifferentiated SCs from their *in vivo* nerve microenvironment, enabling us to define the molecular profile of both states. This revealed several new features of SC reprogramming, including the acquisition of mesenchymal traits. The EMT has emerged as a hallmark of cell fate conversion in development, tumorigenesis and somatic cell reprogramming (Ye and Weinberg, 2015). Our work indicates that natural reprogramming is also accompanied by passage through an EMT-like transition. This finding is further underscored by a recent study on leprosy (Masaki et al., 2013), which demonstrated that the leprosy bacteria reprograms SCs to mesenchymal-like stem cells by downregulating lineage determinants and upregulating EMT genes. Consistent with previous reports, we found SC marker gene expression to be retained in injury-induced dedifferentiation. Indeed, our analyses showed that both bSCs and dSCs remain S100β^+^ and express significant amounts of Sox10 mRNA (Figures S1E and S7D) (Chen et al., 2007, Jessen et al., 2015). Interestingly, however, we found that Sox10 mRNA levels were reduced to similar extents in both dSCs and bSCs compared to iSCs (Figure S7D). Thus, a mesenchymal-like transition is likely not idiosyncratic to leprosy infection, but a physiological response to injury, which is exploited and augmented by the bacteria.

The identification of a partial mesenchymal transition provides important insights into the nature of SC dedifferentiation. Traditionally, dedifferentiation has been considered as a reversal to an earlier developmental stage in the SC lineage (Chen et al., 2007). However, an important study recently challenged this view. The authors demonstrated that dedifferentiated SCs upregulate a specialized repair-promoting transcriptional program orchestrated by c-*jun* and suggested that injury reprograms cells to a “repair cell” (Arthur-Farraj et al., 2012). Our analysis supports this idea and confirms that dedifferentiated SCs are more closely related to ES cells than their developmental progenitors, the neural crest. It also uncovers acquisition of mesenchymal traits as an additional molecular feature of the repair cell. Interestingly, the mesenchymal program was not altered in c-Jun knockout mice, suggesting that other EMT-specific transcription factors are likely involved (Arthur-Farraj et al., 2012). Consistent with this idea, c-*jun* expression was not affected in bSCs and dSCs from *Tgfbr2*^*ΔSC*^ mice (data not shown), whereas the EMT factors Zeb1, Sox9, and HMGA2 were all upregulated in dedifferentiated SCs (Tables S2 and S3) (Lamouille et al., 2014).

The ability to carry out RNA-seq on minute numbers of cells purified from the *in vivo* nerve enabled us to compare the transcriptomes and ES modules of distal and bridge cells. We found that bSCs and dSCs are distinct cell populations with different transcriptomes and repair features, indicating that the microenvironment is a key regulator of the dedifferentiation program. Bridge cells switch off repair genes, increase expression of mesenchymal and Myc target genes, and proliferate more extensively than their distal counterparts. Intriguingly, this transcriptional profile is reminiscent of partially dedifferentiated cancer cells, suggesting that the wound microenvironment may promote a cancer-like phenotype (Kim et al., 2010, Ye and Weinberg, 2015). A link between wounding and cancer is well established in many tissues, including the PNS (Ribeiro et al., 2013, Zhu et al., 2016). Our results reinforce this link and suggest that modulation of cell plasticity may be a key mechanism by which injury sensitizes normal cells to tumorigenesis. They might also explain recent findings that nerve injury induces neurofibroma formation selectively at the wound site in a mouse model of neurofibromatosis type 1 (Ribeiro et al., 2013).

We identified TGFβ as an important mediator of the bSC phenotype. Although TGFβ signaling has been previously linked to the regulation of SC proliferation, apoptosis, and myelination, its role in nerve regeneration had not been previously assessed (D’Antonio et al., 2006; reviewed in Mirsky et al., 2008, Parkinson et al., 2001). We showed that TGFβ signaling is strongly increased across many cell types within the bridge and exploited our conditional, SC-specific *Tgfbr2* knockout model to selectively study its role in bSCs. We found that TGFβ exerts two highly interrelated and synergistic functions in PNS repair that are specific to bridge SCs: it reprograms bSCs to an invasive, mesenchymal-like cell type to drive invasion into the wound, and it concomitantly enhances EphB2-mediated cell sorting to promote directional collective migration. Together, these phenotypes drive repair through the hostile bridge microenvironment to enable peripheral nerve regeneration.

However, consistent with a previous study, TGFβ signaling was dispensable for bSC proliferation, indicating that signals other than TGFβ are responsible (D’Antonio et al., 2006). Our data suggest that TGFβ is also not a key regulator of the increased Myc activity detected in the bridge. Indeed, although TGFβ increased Myc expression *in vitro*, the overlap in differentially expressed Myc target genes between TGFβ-treated SC and bSCs-specific genes was minimal (Tables S2 and S5). It will be important to identify the signals that drive proliferation and Myc within the wound in the future.

Whereas the ability of TGFβ to induce a mesenchymal transition is well established, a crosstalk with Eph signaling has never been observed (Xu et al., 2009). Remarkably, TGFβ not only enhanced cell sorting downstream of EphB2 but was also necessary for EphB2 function. The crosstalk was mediated by the EphB2 effector N-cadherin, which we show is also a TGFβ target. Overall, our mechanistic studies suggest a model whereby TGFβ enhances Eph effects by maintaining high N-cadherin protein levels within bSCs. Upon activation by wound fibroblasts, Eph signaling relocalizes N-cadherin to cell-cell contacts, leading to robust SC sorting and directional migration. In contrast, in the absence of TGFβ signaling, N-cadherin expression decreases to levels that are insufficient for Eph-mediated cell sorting, resulting in disorganized migration of the SCs and their accompanying axons.

In summary, our findings indicate that dedifferentiation is a multifaceted process controlled by the interplay of cell-intrinsic programs and cell-extrinsic signals. Following injury, neuronal degeneration relieves prodifferentiative axonal signals, thereby triggering SC dedifferentiation through activation of cell-intrinsic transcriptional programs. Extrinsic signals from the microenvironment then superimpose on these programs in a context-dependent manner to adapt SC function to the specific repair requirements of their surrounding tissue.

## STAR★Methods

### Key Resources Table

**Table:** 

REAGENT or RESOURCE	SOURCE	IDENTIFIER
**Antibodies**		

Mouse anti-rat OX42	Abcam	Cat# ab33827 RRID: AB_726081
Rabbit polyclonal S100	DAKO	Cat# Z0311 RRID: AB_10013383
Mouse monoclonal N-cadherin	BD Biosciences	Cat# 610920 RRID: AB_2077527
Sheep polyclonal N-cadherin	R and D Systems	Cat# AF6426 RRID: AB_10718850
Goat polyclonal MBP (D-18)	Santa Cruz Biotechnology	Cat# sc-13912 RRID: AB_648794
Chicken polyclonal anti-70kDa neurofilament	Abcam	Cat# ab72997 RRID: AB_1267598
Rabbit polyclonal anti-nerve growth factor (NGF-receptor) p75	Millipore	Cat# AB1554 RRID: AB_90760
Rat monoclonal reticular fibroblast and reticular fibers	Abcam	Cat# ab51824 RRID: AB_881651
Rat monoclonal endomucin (V.7C7)	Santa Cruz Biotechnology	Cat# sc-65495 RRID:AB_2100037
Rabbit monoclonal anti-smad3 (phosho S423 + S425)	Abcam	Cat# ab52903 RRID: AB_882596
Rabbit polyclonal Eph recpeptor B1 + Eph receptor B2 (phosho Y594)	Abcam	Cat# ab61791 RRID: AB_2099832
Goat polyclonal anti-mouse EphB2	R and D systems	Cat# AF467 RRID: AB_355375
Rabbit polyclonal beta actin	Abcam	Cat# ab8227 RRID: AB_2305186
Rabbit monoclonal Smad4 (C-term) clone EP618Y	Millipore	Cat# 04-1033 RRID: AB_1977326
Rabbit polyclonal TGF beta 1	Abcam	Cat# ab25121 RRID: AB_2271652
Moiuse monoclonal TGF beta 2	Abcam	Cat# ab36495 RRID: AB_778343
Rabbit polyclonal anti-smad2 phospho (ser465/Ser467) PhosphoDetect	Millipore	Cat# 566415-50UL RRID: AB_10684669
Mouse monoclonal anti-Smad2	Cell signaling technology	Cat# 3103 RRID: AB_490816
Mouse monoclonal anti-GAPDH	Abcam	Cat# ab9484 RRID: AB_307274
Rabbit polyclonal Sox2	Abcam	Cat# ab97959 RRID: AB_2341193
Goat polyclonal lamin B	Santa Cruz Technologies	Cat# sc-6216 RRID: AB_648156

**Bacterial and Virus Strains**		

Adenoviral cre-GFP	Vector Biolabs	1700
Adenoviral GFP	Vector Biolabs	1060
Adenoviral N-cadherin	Gift from M.Herlyn	N/A

**Chemicals, Peptides, and Recombinant Proteins**		

EdU (5-ethynyl-2′-deoxyuridine)	ThermoFisher	Cat # A10044
Human TGF-β	R and D systems	Cat# 302-B2-002 GenPept: P61812
SD 208	Tocris	Cat# 3269 Cas # 627536-09-8
Galunisertib (LY2157299)	Selleckchem	Cat# S2230 Cas # 700874-72-2
Recombinant mouse Ephrin-B2 Fc Chimera Protein	R and D Systems	Cat# 496-EB Accession# AAA82934
AffiniPure Anti-human IgG, Fc fragment specific	Jackson ImmunoResearch Labs	Cat# 109-005-008 RRID: AB_2337534

**Critical Commercial Assays**		

Click-iT™ EdU Alexa Fluor™ 488 Imaging kit	ThermoFisher	Cat # C10337
Lightning-Link APC conjugation kit	Innova Bioscience	Cat # 705-0005
Nextera XT DNA library preparation kit	Illumina	Cat # FC-131-1096

**Deposited Data**		

Raw and analyzed data	This paper	GEO: GSE103039
Mouse reference genome NCBI Build 37, USCS mm9	UCSC Genome Browser Gateway	http://hgdownload.cse.ucsc.edu/downloads.html
Rat reference genome RGSC Rnor_5.0	Ensembl	http://www.ensembl.org/Rattus_norvegicus/Info/Annotation

**Experimental Models: Cell Lines**		

Primary Rat Schwann Cells isolated at p7	This paper	N/A
Primary Mouse Schwann Cells isolated at p2	This paper	N/A

**Experimental Models: Organisms/Strains**		

Mouse: P0A-cre	RIKEN BioResource Center	RRID: IMSR_RBRC01459
Mouse: B6.Cg-Gt(ROSA)*26Sor*^*tm14(CAG-tdTomato)Hze*^	The Jackson Laboratory	RRID: IMSR_JAX:007914
Mouse Tgfbr2^tm1Karl^	The Jackson Laboratory	RRID: IMSR_JAX:012603

**Oligonucleotides**		

Mouse MPZ primer Fwd: ctttgccctaccccagctatg Rev: acggcaccatagatttccct	This paper	N/A
Mouse Plp1 primer Fwd: cacctgtttattgctgcgtt Rev: cgcacagaccagcaaggatt	This paper	N/A
Moue Pmp22 primer Fwd: actgtaccacatccgccttg Rev: cgcacagaccagcaaggatt	This paper	N/A
Mouse Sox2 primer Fwd: catgggctctgtggtcaagt Rev: tacatggtccaattcccccg	This paper	N/A
Mouse cJun Primer Fwd: agcagacgcttgagttgaga Rev: gggtccctgcttttgagataa	This paper	N/A
Mouse Ngfr Primer Fwd: agctcccagcctgtagtgac Rev: gcagctgttccatctcttga	This paper	N/A
Mouse Tgfβ2 Primer Fwd: gcagctgttccatctcttga Rev: gctggactgttgtgactcca	This paper	N/A
Mouse Cdh2 Primer Fwd: tgtgggaaagatcaagtcca Rev: ttccctgtgttagcatcgac	This paper	N/A
Mouse Fstl3 Primer Fwd: aatcagcctgctagggttcc Rev: ctccgtcgcaggaatcttt	This paper	N/A
Mouse Tagln Primer Fwd: ccttccagtccacaaacgac Rev: gggccagaggagtcatcc	This paper	N/A
Mouse Ctgf Primer Fwd: cgccaagcctgtcaagtt Rev: ccgcagaacttagccctgta	This paper	N/A
Mouse Cyr61 Primer Fwd: cccttctccacttgaccaga Rev: cacttgggtgcctccaga	This paper	N/A
Mouse Plaur Primer Fwd: gtgttgcaactacacccactg Rev: cacttgggtgcctccaga	This paper	N/A
Mouse Pmepa1 Primer Fwd: tcatgcagaagcgggttag Rev: cagggtggaactgcgtaga	This paper	N/A
Mouse Edil3 Primer Fwd: gaaggcattgtactttaagaatgga Rev: tttcatccccaaaggttctg	This paper	N/A
Mouse Tpm4 Primer Fwd: cctggagggtgagctgaa Rev: tcttccaggtcaccacacttt	This paper	N/A
Mouse Adam12 Primer Fwd: cagagagtttcagaggcaagg Rev: gcgatctctattaatcgctgct	This paper	N/A
Mouse Fbln5 Primer Fwd: tgatatggacgagtgcagctt Rev: ggctggttcacacactcgt	This paper	N/A
Mouse Plod2 Primer Fwd: tccaaactcatggacacagg Rev: tgtaaccttgggagggacat	This paper	N/A
Mouse Pdlim4 Primer Fwd: tccacattgaccctgagtcc Rev: cctccagactaatcccagagac	This paper	N/A
Mouse Postn Primer Fwd: cgtggaaccaaaaattaaagtcat Rev: cttcgtcattgcaggtcctt	This paper	N/A
Mouse Tnc Primer Fwd: gatgccacctcccatgtc Rev: gcccttctcagcaatgagg	This paper	N/A
Mouse Lrrc15 Primer Fwd: ggggaagcaaaattgctaaga Rev: ggtttccattacacttggtcgt	This paper	N/A
Mouse Gja1 Primer Fwd: gagagcccgaactctccttt Rev: cgccaaagtggtggaact	This paper	N/A
Mouse Sdc1 Primer Fwd: ggaagtgctgggaggtgtca Rev: gaaagccaccaggcacacag	This paper	N/A
Mouse Mcm7 Primer Fwd: gcttttgcctgagtacaagga Rev: tcagccggtgttcaatgtaa	This paper	N/A
Mouse Timp1 Primer Fwd: gcaaagagctttctcaaagacc Rev: agggatagataaacagggaaacact	This paper	N/A
Mouse Lgals1 Primer Fwd: ctcaaagttcggggagaggt Rev: cattgaagcgaggattgaagt	This paper	N/A
Mouse Dyps13 Primer Fwd: tggggtgggacgtgtact Rev: acacacaatgcccttctgc	This paper	N/A
Mouse B2M Primer Fwd: ctgctacgtaacacagttccaccc Rev: catgatgcttgatcacatgtctcg	This paper	N/A
Mouse Tgfbr2 Exon4 Primer Fwd: acgttcccaagtcggatgtg Rev: ttcagtggatggatggtcct	This paper	N/A
Rat Crlf1 Primer Fwd: ggatgtcctggacgtggt Rev: gagacccagcgcactc	This paper	N/A
Rat Fstl3 Primer Fwd: ggtgctgaagacacaggtca Rev ggaatcctagcaggctgattt	This paper	N/A
Rat Tagln Primer Fwd: tccagactgttgacctctttga Rev: actgcccaaagccattacag	This paper	N/A
Rat Cyr61 Primer Fwd: aggtgtttcaaaactgccgta Rev: actgcgactgcgttactgt	This paper	N/A
Rat Adam12 Primer Fwd: tcttccctgaaccctctcag Rev: tggtcatcttaagggtctctcttt	This paper	N/A
Rat CD44 Primer Fwd: gagaaaactggacccaggaac Rev: ttaggatctgcccaggttgt	This paper	N/A
Rat Tpm4 Primer Fwd: ggcggaggtgtctgaactaa Rev: tcagattgttagttacgttcttgagc	This paper	N/A
Rat Fbln5 Primer Fwd: cacggtgggctcctacac Rev: ctgtctcacactcattcactcc	This paper	N/A
Rat Edil3 Primer Fwd: tcgaaggcactgtacattaagaat Rev: tttcatccccaaggttctg	This paper	N/A
Rat Tnfsrsf12a Primer Fwd: ttcgggttggtgttgataca Rev: ccagtctcctctatgggggta	This paper	N/A
Rat Plod2 Primer Fwd: ctaaacacgacatcagctctataaaaa Rev: atcctgacggcagaaatcc	This paper	N/A
Rat Pdlim4 Primer Fwd: ggttcatgctgggagcaa Rev: gcttggatcgagtcacctg	This paper	N/A
Rat Cdh2 Primer Fwd: cccacttacggcctttca Rev: gtaaactctggaggattgtcattg	This paper	N/A
Rat B2M Primer Fwd: cgctcggtgaccgtgatctt Rev: cggtggatggcgagagtaca	This paper	N/A
siRNA targeting sequence Smad4 (oligo1) 5′-CAGCTACTTACCACCATAACA-3′	QIAGEN	SIO3113068
siRNA targeting sequence Smad4 (oligo2) 5′-TCAGTAGCATTTGACTTAAA-3′	QIAGEN	SIO1905232
siRNA targeting sequence Cdh2 5′-CAGCGCGGTCTTACCGAAGGA-3′	QIAGEN	SIO1497230

**Software and Algorithms**		

TopHat v.2.0.11	Kim et al., 2013b	https://ccb.jhu.edu/software/tophat/index.shtml
HTSeq v.0.6.1	Anders et al., 2015	http://htseq.readthedocs.io/en/release_0.9.1/overview.html
The R project		https://www.r-project.org/
DESeq2 Bioconductor package	Love et al., 2014	http://bioconductor.org/packages/release/bioc/html/DESeq2.html
GOplot R package	Walter et al., 2015	https://cran.r-project.org/web/packages/GOplot/index.html
GSEA	Subramanian et al., 2005	http://software.broadinstitute.org/gsea/index.jsp
DAVID	Walter et al., 2015	https://david.ncifcrf.gov/

### Contact for Reagent and Resource Sharing

Further information and requests for resources and reagents should be directed to and will be fulfilled by the Lead Contact, Dr Simona Parrinello (simona.parrinello@lms.mrc.ac.uk).

### Experimental Model and Subject Details

#### Animals

Mice were bred and maintained in the animal care facility at Hammersmith Hospital. All animal procedures were carried out in accordance with the Animal Scientific Procedures Act, 1986 and in accordance with the local ethical and care guidelines at Imperial College London, Hammersmith Hospital and the International guidelines of the Home Office (UK). Mouse lines were obtained from the Jackson Laboratory and the Riken BioResource. In order to specifically identify Schwann cells *tdTom*^*SC*^ reporter mice were generated by crossing the protein myelin zero promoter specific cre recombinase mice P0A-Cre (Giovannini et al., 2000) obtained from RIKEN BioResource Center (RBRCo1459) to B6.Cg-Gt(ROSA)26Sor^*tm14(CAG-tdTomato)Hze*^ mice (Madisen et al., 2010) obtained from The Jackson Laboratory (Jax 007914), which express tdTomato after removal of a floxed stop codon under the control of the Rosa promoter. In line with previous reports (Giovannini et al., 2000), Cre-mediated recombination led to 70%–95% of both myelinating and nonmyelinating Schwann cells expressing tdTomato as assessed by staining of 10 μm *tdTom*^*SC*^ nerve cryosections with with Myelin basic protein and p75^NGFR^ antibodies, respectively (Figures S1A and S1B). Deletion of TGFβ signaling in nerves was achieved by crossing *P0A-Cre* mice with *Tgfbr2*^*tm1Karl*^ mice (Levéen et al., 2002) obtained from The Jackson Laboratory (Jax 012603), which possess loxP sites flanking exon 4 of the *Tgfbr2* gene to generate *Tgfbr2*^*ΔSC*^ animals. Deletion of exon 4 leads to a loss in TGFβ signaling in 50%–85% of Schwann cells as assessed by genomic PCR and phospho-smad3 staining in the nerves (Figures S4C and S4D). *Tgfbr2*^*ΔSC*^ animals were also crossed to B6.Cg-Gt(ROSA)26Sor^*tm14(CAG-tdTomato)Hze*^ reporter mice to identify recombined cells. 70%–90% of tdTomato^+^ Schwann cells were found to also carry *Tgfbr2* recombination in these animals.

#### Sciatic nerve transections

Full sciatic nerve transections were carried out on male and female mice aged 6-8 weeks. The left sciatic nerve was exposed under isoflurane general anesthesia in aseptic conditions and transected at a point 1cm from the sciatic notch. The incision was closed using surgical clips and vetergesic analgesia was administered. Six days post-operation transected nerves, along with the contralateral control nerve, were collected for subsequent studies. For proliferation studies, mice were injected intraperitoneally with EdU (50mg/kg; Invitrogen) 4 hr prior to nerve collection. For the time course study nerves were collected at d1, d2, d4, d6, d8 and d10 post-transection.

#### Schwann Cell Cultures

Primary rat Schwann cells were prepared from p7 Sprague-Dawley rats (male and female). Sciatic nerves were dissected from pups, finely chopped and digested in EBSS containing DNase (7 units), collagenase (1mg/ml) and 0.025% trypsin at 37°C for 35 min. Macrophages were removed from digested cells by panning with anti-CD11 antibody OX42 (Harlan sera-labs, MAS 370p) at 1:500 dilution. Fibroblasts were removed from the macrophage depleted cells by panning three times using Thy1 antibody (OX7 1:1000 BD Biosciences 554898). Rat Schwann cells were plated onto poly-L-lysine (1:2500) and laminin (1:100) coated dishes in Schwann cell media (DMEM, GIBCO 11880-028) containing 3% FCS, 4mM glutamine, 1 μM forskolin, kanamycin/gentamycin and GGF (Glial growth factor, manufactured in house).

Mouse Schwann cells were prepared from *Tgfbr2*^*fl/fl*^ pups (male and female) at post-natal day 2. Sciatic and brachial nerves were collected, digested in a 1:1 mixture of 0.4% collagenase type 2 (Worthington 405U/mg-4176) and trypsin 1:300 (GIBCO 840-7073 2.5mg/ml) in tilted 35 mm dish at 37C for 30-35 min. Cells were pelleted, suspended in dissociation medium (DMEM low glucose from GIBCO 31885-023, 5% horse serum, arabinosylcytosine and penicillin/streptomycin and plated on poly-L-lysine (1:2500) and laminin (1:100) coated dishes. After 3 days cells media was replaced with complete G5 medium: Neurobasal media (ThermoFisher 21103049) containing 4mM glutamine, G5 supplement (ThermoFisher 17503012), heregulin (20ng/ml PreProtech 100-03), dibutryl-cyclic AMP (0.1mM Sigma D0260), kanamycin/gentamycin and 3% horse serum.

### Method Details

#### Preparation of mouse Schwann cells for FACS and RNA extraction

Transected nerves, or contralateral control nerves, from *tdTom*^*SC*^ mice (male and female) were collected into ice-cold HEPES buffered HBSS. The bridge region, and the nerve portion distal to the wound were separated from the proximal nerve by dissection under a fluorescent stereomicroscope. In each dissection 90mm of nerve distal to the wound site was collected. For characterization of the SC transcriptome shown in Figures 1, 2A–E, and 7F, nerves from 3 mice were pooled for each FACS sample. This yielded ≥ 250pg RNA/sample enabling the generation of high coverage sequencing data. Bridges from 3 mice were digested together while the distal and intact nerves were digested separately and pooled prior to centrifugation. Nerve fractions were finely chopped prior to digestion in a trypsin (GIBCO 1:250 3mg/ml): collagenase II (Worthington 1.62U/ul): hyaluronidase (Worthington 1%) mix 1:1:0.04 with the addition of pronase (Sigma-Aldrich 10%) 1 μl/50 μl. Nerves were digested for a total of 20 min at 37°C with trituration after 10 min. Digestion was terminated by trituration in dissociation medium (DMEM containing 5% horse serum and penicillin-streptomycin). Following centrifugation (10 min, 1000rpm, 4°C) cells were resuspended in 400 μL FACs sorting buffer containing 2.5% RNAsin (Promega). tdTomato-expressing cells were FACs sorted (BD FACS ARIA III machine) and collected into RLT buffer (QIAGEN) along with non-tdTomato-expressing cells and RNA was extracted using the RNeasy Plus Micro Kit according to manufacturer’s recommendations (QIAGEN). For time-course analysis, RNA was extracted from single intact nerves as control, distal nerve stumps at d1, d2 post-transection (no bridge is present at this stage) and both bridge and distal regions at d4, 6, 8 and 10.

#### Characterization of FACS sorted cells

FACS sorted cells were collected into 350μl of dissociation buffer which was transferred to a PLL coated 4 well plate in complete Schwann cell media (Parrinello et al., 2010). The cells were allowed to adhere for 6 hr to overnight (37°C with 10% CO_2_) and characterized by immunofluorescence for S100β expression to determine purity of the Schwann cell preparation, or were stained with Calcein AM (1:10,000 for 10 min, Sigma), to assess cell viability of the tdTomato^+^ cells. For quantitative assessment of purity, dissociated tdTomato^+^ Schwann cells were acutely stained with S100-APC linked (1:50; Lightning-Link Innova Biosciences) and subjected to FACS analysis to assess coexpression of S100β and tdTomato.

#### RNA-Sequencing Library Preparation

RNA libraries from FACS sorted cells were prepared from samples which had an RNA integrity number (RIN) greater than 7 and were sufficiently concentrated to provide a minimum of 250pg RNA in a maximum volume of 2.4 μl. Samples were spiked with ERCC spike-in controls (Mix 1 ThermoFisher) at a dilution of 1:1x10^6^. dsDNA libraries were prepared according to the Smart-seq2 protocol (Picelli et al., 2014) and 1ng was tagmented using the Nextera XT DNA library preparation kit (Illumina) according to manufacturer’s instructions. The libraries were diluted to a final concentration of 2nM, the samples pooled and 10pmol were sequenced on an Illumina HiSeq 2500 instrument. Time course RNA libraries were prepared from a minimum of 100pg of RNA with a RIN greater than 7.

Rat Schwann cell libraries were prepared from RNA extracted using the Trizol method from control Schwann cells or Schwann cells treated overnight with 10ng/ml recombinant TGFβ (R&D Systems). 500ng of RNA was used for library preparation using the NEB Next Ultra Directional RNA library prep kit for Illumina (NEB). RNA sequencing was performed on pooled libraries as above.

RNA sequencing data was processed using RTA version 1.18.64, with default filter and quality settings. The reads were demultiplexed with CASAVA 1.8.4 (allowing 0 mismatches). Raw reads were aligned to mouse (NCBI Build 37, USCS mm9) or rat (RGSC Rnor_5.0) genomes using the TopHat v.2.0.11 software (Kim et al., 2013b) and assigned to genomic features using HTSeq v.0.6.1 (Anders et al., 2015). RNA-seq data derived from single nerves were preprocessed by i) removing datasets containing less than 10000 detectable genes, ii) filtering out genes with less than 100 raw counts in more than 15% of the remaining datasets. After filtering, 4 intact single nerves and 3 of each other conditions were included in our analysis, each containing data for > 6500 genes (Table S6). Differential expression, RPKM, and normalized counts where generated using the DESeq2 Bioconductor package (Love et al., 2014). Figures 1E and 3C are based on normalized counts and 2B on preranked ratios. Downstream data analysis was performed in R. Functional analysis was performed using the DAVID and GSEA toolsets (Huang da et al., 2009, Subramanian et al., 2005). Visualization of GO enrichments on Figure 1D was generated using the GOplot R package and outputs from DAVID (Walter et al., 2015). Visualization of GSEA enrichment on Figure 2B was generated using R code made available from https://www.biostars.org/p/168044/. ES modules were described in (Kim et al., 2010). Figure 1F represents the mean expression values in FPKM from the iSC, dSC, and bSC datasets, mature astrocytes and neurons from (Zhang et al., 2014) and embryonic stem cells from (Marks et al., 2012). FPKM from all datasets were further normalized by median centring to allow cross-comparison.Schwann cell TGFβ signaling signatures were defined by DESseq2 analysis of RNA-seq data from rat Schwann cells treated *in vitro* with TGFβ. Signatures include genes up or downregulated more than 2 fold with an adjusted p value < 0.0001 (Table S5) (Love et al., 2014). The list of 200 genes form the HALLMARK_EPITHELIAL_MESENCHYMAL_TRANSITION available on the GSEA website was used as EMT signature.

#### Cell Clustering Experiments and Infections

For clustering experiments, rat or mouse Schwann cells (2.8x10^4^) were plated on PLL-treated coverslips which had been coated with recombinant ephrinB2-Fc clustered with anti-Fc antibodies at a 2:1 molar ratio (8ug/ml R&D Systems) at 37°C overnight or with molar equivalent anti-human Fc antibodies. Cells were plated in the presence or absence of 10ng/ml recombinant TGFβ (R&D Systems) and 16 hr later fixed and stained for S100β (DAKO ZO311; 1:500) and N-cadherin (BD Biosciences 610921; 1:500). Quantification of cell sorting was performed on duplicate coverslips. A minimum of 200 cells per coverslip was counted across randomly selected fields of view. Two coverslips were counted for each condition. Cells were assigned to groups depending on whether they were single, in a cluster of 2-9 cells or in a cluster of more than 9 cells and percentage of cells in clusters of increasing size calculated. N-cadherin at cell-cell contacts was measured in ImageJ.

Samples for western blotting were plated at a density of 1.25 × 10^5^ in 6-well plates previously coated with ephrinB2-Fc or Fc as above in the presence or absence of 10ng/ml recombinant TGFβ and collected after 16 hr in RIPA buffer. For inhibition of TGFR activity cells were cultured in the presence or absence of two independent inhibitors; LY2157299 (Selleck; final concentration 5nM) and SD208 (Tocris Bioscience; final concentration 2.5nM).

siRNA transfection of Schwann cells were performed For siRNA oligonucleotides were transfected using Hiperfect according manufacturer’s instructions (QIAGEN). Silencing target sequences for *Smad4* were: 5′-CAGCTACTTACCACCATAACA-3′ (oligo 1) and 5′-TCAGTAGCATTTGACTTAAA-3′ (oligo 2), oligonucleotides were used at a final concentration of 25nM. For *Cdh2* target sequence was 5′-CAGCGCGGTCTTACCGAAGGA-3′ and was used at a final concentration of 4nM to produce partial knockdown.

N-cadherin rescue in rat Scwhann cells and deletion of *Tgfbr2* in mouse cells was achieved by adenoviral infection. *Tgfbr2*^*fl/fl*^ mouse cells were transfected with adenoviruses encoding Cre recombinase. Control cells were infected with adenoviruses encoding GFP (Ad-*Gfp*). Adenoviral supernatants were prepared as described in the manufacturer’s instructions (Vivapure-VivaScience) and added at a multiplicity of infection of ?80 to Schwann cells. Twenty-four hours later the adenoviral supernatant was removed, and the medium changed to fresh growth medium. Infection conditions were optimized to achieve maximal infection efficiency as judged by the percentage of GFP-positive cells (> 95%) and minimal cell toxicity. Successful *Tgfbr2* deletion was confirmed by qPCR (Figure S4I).

#### Invasion Assays

Invasion of rat Schwann cells (2x10^5^) through a 1:40 fibronectin matrix was carried out using the xCelligence system and CIM-Plates 16 (ACEA Biosciences) according to manufacturer’s instructions. Cells were cultured overnight in the presence or absence of 10ng/ml TGFβ prior to seeding and invasion was allowed to proceed for 48 hr. Measurements were taken at the point of maximal invasion, which corresponded to 4-6 hr after seeding.

For invasion of mouse Schwann cells, 5x10^5^ cells were placed in the upper well of Boyden Chamber (Millicel 8.0 μm pore size) in the presence or absence of TGFβ (10ng/ml). Invasion through fibronectin-coated (1:40) membrane was allowed to proceed for 48 hr before cells were fixed, stained with dapi (1:5000) and counted.

#### Immunofluorescence, Immunohistochemistry and Western Blotting

For quantifications of axonal regrowth and Schwann cell migration (Figure 4), cryo-sections (50 μm) of cut sciatic nerves were processed for immunohistochemistry. Sections were thawed (10 min at room temperature), post-fixed (15 min 4% paraformaldehyde), treated with NH_4_Cl_2_/glycine (0.2%/0.37%) before permeabilisation (3 hr in 1%NP-40 in TBST; tris-buffered saline with 0.1% tween 20). Sections were blocked and antibdodies added in TBST containing 10% donkey serum). All other immunostainings were carried out on 15 μm cryo-sections. Antibodies were: anti-S100β (DAKO ZO311; 1:1000), chicken polyclonal anti-neurofilament (Abcam ab72997 1:2000), sheep polyclonal anti-N-cadherin (R&D systems AF426 1:200), Myelin basic protein (MBP, Santa Cruz sc-13912 1:100) and p75^NGFR^ (Millipore ab1554 1:1000). In addition, 5 μm paraffin sections were processed for phospho-smad3 (Abcam ab52903 1:50) and S100β (DAKO ZO311 1:500) staining. To confirm the identity of psmad3 stained cells within the wound site psmad3/S100β stained sections were also stained for fibroblasts (reticular fibroblast antibody1:500 ab51824) and endothelial cell markers (endomucin 1:1000 sc65495). For identification of EdU incorporation 15 μm cryostat sections were stained using the Click-It kit (Invitrogen) according to manufacturer’s instructions.

Western blotting and immunostaining of cells were performed according to standard protocols. Antibodies used for western blotting were as follows: pEphB1/B2, rabbit polyclonal (Abcam ab61791 1:1000); EphB2, goat polyclonal (R&D Systems AF467 1:2000); β-actin rabbit polyclonal (Abcam ab8227 1:5000); N-cadherin mouse monoclonal (BD Biosciences 610921; 1:1000); Smad4, rabbit polyclonal (Millipore 04-1033; 1:1000). TGFβ1 – rabbit polyclonal (1:1000 Abcam ab25121); TGFβ2 – mouse polyclonal (1:1000 Abcam ab36495): phospho-smad2 – rabbit polyclonal (1:1000 Merck 566415); smad2 – mouse monoclonal (1:1000 Cell Signaling Technology 3103S); gapdh – mouse monoclonal (1:10,000 Abcam ab9484): Sox2 – rabbit polyclonal (1:1000 Abcam ab97959); laminB – goat polyclonal (1:1000 Santa Cruz sc-6216).

#### Quantitative RT-PCR

RNA was extracted from control Schwann cells or from Schwann cells treated overnight with 10ng/ml TGFβ (R&D systems) using Trizol Reagent (Ambion). Frozen nerve samples were homogenized in Trizol Reagent using a TissueRuptor (QIAGEN). RNA was reverse transcribed using iScript gDNA clear cDNA synthesis kit (Bio-rad) and quantitative PCR was performed using the qPCRBIO SyGreen Mix Lo-Rox (PCR Biosystems). For quantitative RT-PCR from bridge and distal FACS-purified Schwann cells prepared from transected nerves of *Tgfbr2*^*fl/fl*^ *or Tgrb2*^*ΔSC*^ mice, cDNA libraries were prepared according to the Smart-Seq2 protocol. RNA input was 200pg for distal cells and 100pg for the bridge cells. Relative expression values for each gene of interest were obtained by normalizing to B2M and plotted in R.

#### In vivo quantifications

Quantifications of Schwann cell migration and axonal regrowth were carried out on fluorescent images acquired using inverted SPE confocal microscopes (Leica). 50 μm cryosections from *Tgfbr2*^*fl/fl*^ *and Tgfbr2*^*ΔSC*^ nerves were stained with S100β and/or neurofilament and DAPI and for each nerve, the same volume and number of z- stacks was taken and the same settings of acquisition were used. A projection of the z stacks was made and used for quantifications using Fiji software (http://fiji.sc/Fiji). For quantifications at 6 days post-transection, images of the 4 central sections of the bridge region of each nerve were taken and analyzed, as follows. A horizontal line was drawn demarcating the boundary between proximal stump and bridge, as assessed by DAPI staining and Schwann cell morphology. Lines perpendicular to this boundary were then drawn from the leading edge of each Schwann cord to the boundary itself across the entire width of the nerve. Cord length was then averaged in each mouse to calculate ‘average cord length’. Maximum migrated distance was also measured as the longest cord in each animal. For quantification of axonal regrowth at 6 weeks post-transection, z stack images of distal stumps stained for neurofilament to identify axons were taken across the entire width of the nerve. Projections were converted to binary images and area occupied by axons was measured as a readout of reinnervation. 3 areas of identical size were measured along the entire length of the distal stump at regular intervals from the wound site and average axonal density calculated for each mouse. Quantifications of cell sorting were carried out in *tdTom;Tgfbr2*^*fl/fl*^ and *tdTom;Tgfbr2*^*ΔSC*^ mice to facilitate visualization of bridge Schwann cells. Images of the proximal bridge were taken across the four sections encompassing the central region of the nerve. Within each section, two areas of identical size were selected proximal to the leading edge of the Schwann cell cords and number of Schwann cells in cords in each area quantified and expressed as percentage of total number of cell per area.

#### Electron microscopy and morphological analyses

Sciatic nerves were collected and fixed in 2% (vol/vol) glutaraldehyde in 0.12M phosphate buffer, post-fixed in 2% osmium tetroxide overnight at 4C and in 2% Uranyl Acetate 45 min at 4C. Nerves were then dehydrated in an ethanol series before embedding in epoxy resin.

Semithin sections were cut with a glass knife at 0.1 μm, dried carefully and stained with 0.5% toluidine blue in 2% borax at 75°C for 30 s. After dehydration, sections were mounted with DPX (Sigma) and representative images were acquired using wide-field microscopy.

Ultrathin sections were cut with a diamond knife at 70 nm, collected onto formvar coated slot grids, stained with lead citrate and then visualized using transmission electron microscopy (T12 Tecnai Spirit, FEI) using a Morada camera and iTEM software (Olympus SIS).

*g* ratios were determined by dividing the mean diameter of an axon without myelin by the mean diameter of the same axon with myelin. To determine the size distribution of myelinated fibers, axon diameter of 9 images (three 75.58x50.34 microns fields per mouse, 3 mice per genotype) were measured and binned based on their diameter. All measurements were acquired using Photoshop to draw the axons and their associated myelin sheath and Fiji software to measure their mean diameter.

### Quantification and Statistical Analysis

Statistical analysis was performed using Prism (GraphPad Software). Fisher’s exact test was used for the clustering experiments; Student’s t test was used for paired data and ANOVA with Bonferroni correction for all group data. All experiments for which quantifications were performed were carried out a minimum of three times as indicated in the figure legends. Data are presented as mean ± SEM p < 0.05 was considered significant.

### Data and Software Availability

Gene expression data are available in the Gene Expression Omnibus, GEO: GSE103039. Freely available software and algorithms used for analysis are listed in the resource table. Some unavailable analyses were specifically designed for the purpose of this paper using routines written in R R version 3.3.1 (https://www.r-project.org/). All custom scripts and data contained in this manuscript are available upon request from the Lead Contact.

## Author Contributions

Conceptualization, S.P., M.P.C., and S.M.; Methodology, M.P.C. and E.B.; Software, S.M., S.K., and L.F.C.G.; Investigation, M.P.C., E.B., A-L.C., L.Z., A.A., and J.J.B.; Writing – Original Draft, S.P.; Writing – Review & Editing, S.P., M.P.C., S.M., and A.C.L.; Funding Acquisition, S.P., S.M., and A.C.L.; Resources, A.C.L.; Visualization, S.P., M.P.C., and S.M.; Supervision, S.P. and S.M.

## Supplemental Information

Document S1. Figures S1–S7

Table S1. Sample Definition and Sequencing Statistics, Related to Figures 1, 2, and 3

Table S2. DESeq2 Differential Expression Analysis and Normalized Counts, Related to Figure 1 and 2

Table S3. Expression Data in RPKM, Related to Figure 1 and 2

Table S4. Genes from 7 K-Means Clusters of D/I, B/I, and B/D Expression Ratios, Related to Figure 2

Table S5. Transcriptional Signatures of Rat Schwann Cells Treated with TGFβ In Vitro, Related to Figure 3

Table S6. Time Course Analysis Dataset, Related to Figure 2 and 3

Document S2. Article plus Supplemental Information
