# Supplementary figures and images for: The Wound Microenvironment Reprograms Schwann Cells to Invasive Mesenchymal-like Cells to Drive Peripheral Nerve Regeneration
Source: Neuron. 2017 Sep 27;96(1):98–114.e7. doi: 10.1016/j.neuron.2017.09.008 (PMC5626803; doi:10.1016/j.neuron.2017.09.008)

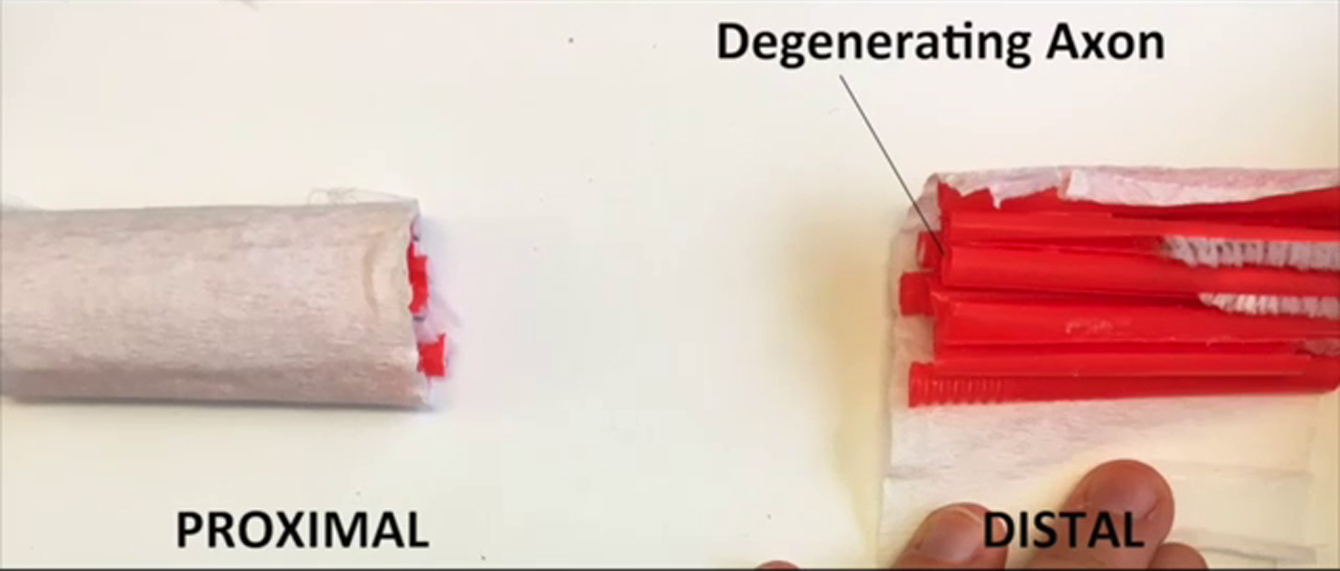

Supplement: Supplementary file 1 [file mmc9.jpg]
