# Supplementary material for: The Wound Microenvironment Reprograms Schwann Cells to Invasive Mesenchymal-like Cells to Drive Peripheral Nerve Regeneration
Source: Neuron. 2017 Sep 27;96(1):98–114.e7. doi: 10.1016/j.neuron.2017.09.008 (PMC5626803; doi:10.1016/j.neuron.2017.09.008)
Supplement: Document S1. Figures S1–S7 [file mmc1.pdf]

Neuron, Volume *96*

Supplemental Information

The Wound Microenvironment Reprograms Schwann Cells to Invasive Mesenchymal-like Cells to Drive Peripheral Nerve Regeneration

Melanie P. Clements, Elizabeth Byrne, Luis F. Camarillo Guerrero, Anne-Laure Cattin, Leila Zakka, Azhaar Ashraf, Jemima J. Burden, Sanjay Khadayate, Alison C. Lloyd, Samuel Marguerat, and Simona Parrinello

Figure S1

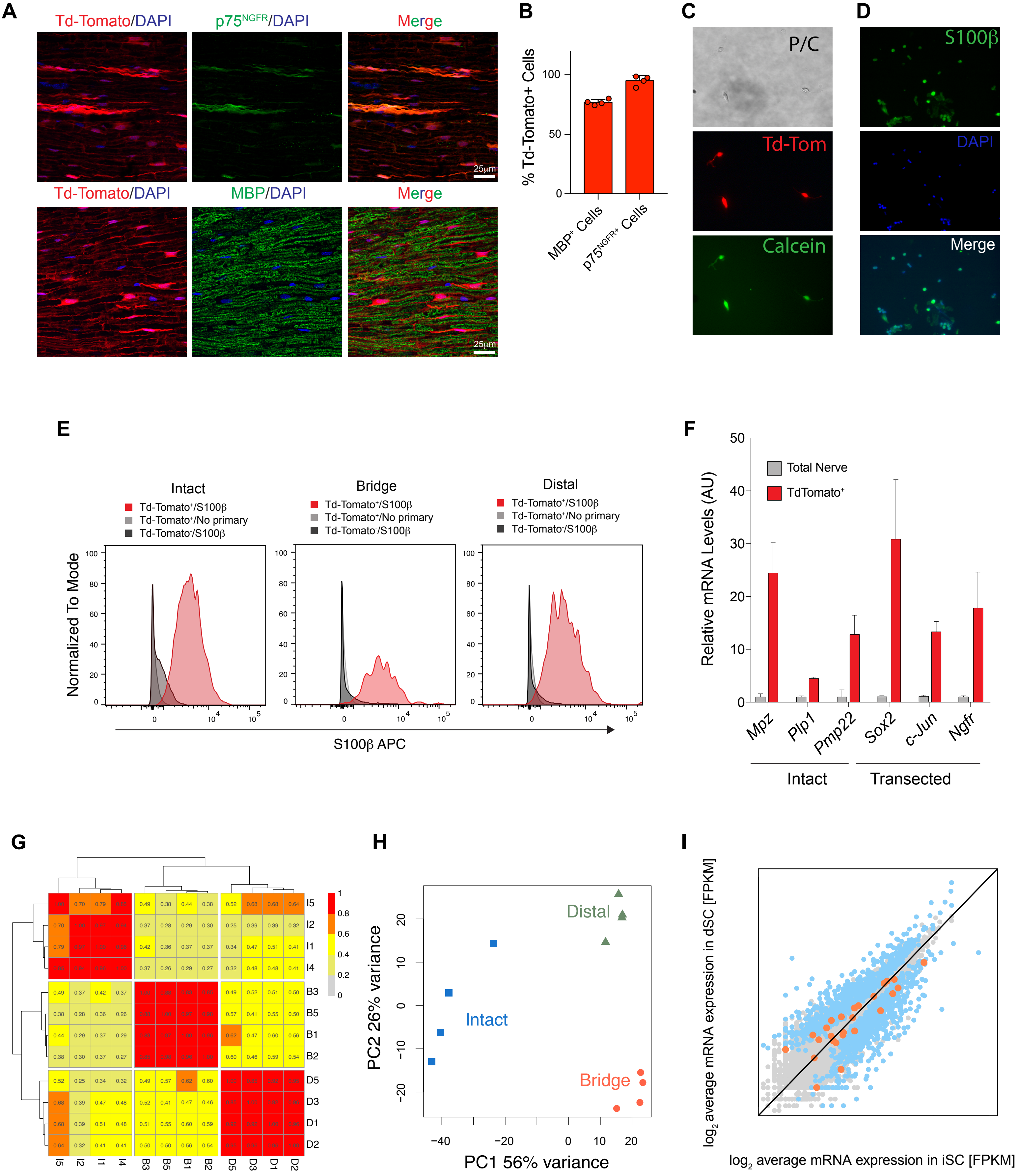
A
Td-Tomato/DAPI
p75NGFR/DAPI
Merge
Td-Tomato/DAPI
MBP/DAPI
Merge
25µm
25µm
B
% Td-Tomato+ Cells
100
50
0
MBP+ Cells
p75NGFR+ Cells
C
P/C
Td-Tom
Calcein
D
S100β
DAPI
Merge
E
Intact
Bridge
Distal
Td-Tomato+/S100β
Td-Tomato+/No primary
Td-Tomato-/S100β
Normalized To Mode
S100β APC
F
Relative mRNA Levels (AU)
Total Nerve
TdTomato+
Mpz
Plp1
Pmp22
Sox2
c-Jun
Ngfr
Intact
Transected
G
I5
I2
I1
I4
B3
B5
B1
B2
D5
D3
D1
D2
H
PC1 56% variance
PC2 26% variance
Distal
Intact
Bridge
I
log2 average mRNA expression in dSC [FPKM]
log2 average mRNA expression in iSC [FPKM]

Figure S1. Related to Figure 1

Schwann cell specificity of TdTomato expression and validation of FACS-sorting strategy

(A) Representative longitudinal cryosections of adult sciatic nerves of *tdTom*^{SC} mice stained for p75^{NGFR} (green; top panel) and DAPI (blue) to identify non-myelinating Scwhann cells, and Myelin basic protein (green; bottom panel) and DAPI (blue) to identify myelinating Schwann cells.

(B) Quantification of images from (A) showing recombination efficiency of the tdTomato allele in both myelinating (MBP) and non-myelinating (p75^{NGFR}) Schwann cells. Recombination efficiency is expressed as percentage of total tdTomato^{+} cells (mean ± SEM). n=4.

(C) Images of FACS-purified tdTomato^{+} Schwann cells purified from intact sciatic nerves of *tdTom*^{SC} mice, plated in Schwann cell media for 6h and labelled with calcein AM. Phase contrast (P/C), tdTomato fluorescence and Calcein fluorescence (green) are shown, as indicated. Incorporation of Calcein confirms cell viability.

(D) Images of tdTomato^{+} Schwann cells FACS-sorted from intact sciatic nerves, plated in Schwann cell media for 6h, fixed and stained with S100β (green) and DAPI (blue).

(E) FACS analysis of S100β expression in cell suspension prepared from intact, bridge (day 6) and distal (day 6) *tdTom*^{SC} nerves. Histograms show the relative fluorescence intensity of tdTomato^{+} Schwann cells stained with anti-S100β-APC linked antibodies (tdTomato^{+}/S100β, red), control unstained tdTomato^{+} Schwann cells (tdTomato^{+}/no primary, light grey) and tdTomato^{-} nerve cells stained with anti-S100β-APC linked antibodies (tdTomato^{-}/S100β, dark grey). In each fraction (intact, distal and bridge) the vast majority of tdTomato^{+} cells are also S100β^{+}, indicating that the FACs sorted cells are indeed Schwann cells. In contrast a negligible fraction of tdTomato^{-} cells are S100β positive.

(F) Gene expression levels of differentiation (*Mpz, Plp1, Pmp22*) and dedifferentiation markers (*Sox2, c-jun, Ngfr*) measured by RT-qPCR in FACS-purified Schwann cells and whole intact and injured nerves. Levels are expressed as fold change relative to whole intact nerves for differentiation markers and to distal stump of injured nerves for dedifferentiation markers. Data are represented as mean ± SEM, n=4.

(G) Hierarchical clustering of Pearson correlations of DESeq2 normalised counts for the *in vivo* RNA-seq libraries from day 6 nerves included in this study. I, B and D stand for intact, bridge and distal Schwann cells, respectively. Colour represent Pearson correlation coefficient and actual values are displayed on their corresponding heatmap tiles.

(H) PCA analysis of DESeq2 expression scores for the *in vivo* RNA-seq libraries (day 6) included in this study after normalisation by variance stabilisation transformation.

(I) Comparison of the $\log_2$ average gene expression scores in FPKM of the 4 libraries obtained from intact (iSC) and distal (dSC) Schwann cells, as indicated. Genes differentially regulated with adjusted $p<0.05$ after DESeq2 analysis are highlighted in light blue. Average expression of ERCC RNA controls spiked in at constant concentration in each libraries are highlighted in orange.

Figure S2

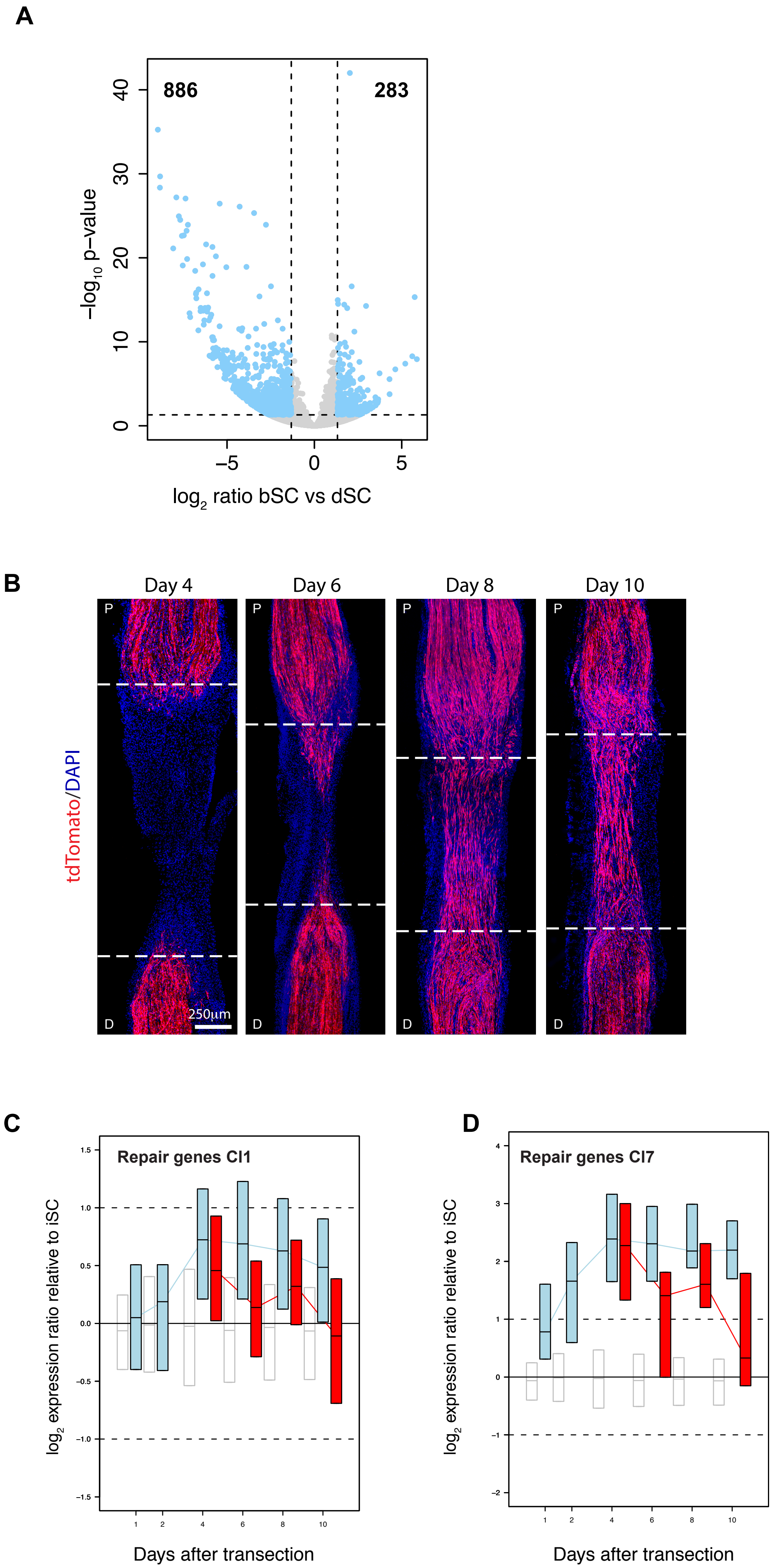
A
886
283
−log10 p−value
log2 ratio bSC vs dSC
B
Day 4
Day 6
Day 8
Day 10
P
D
tdTomato/DAPI
250μm
C
Repair genes Cl1
log2 expression ratio relative to iSC
Days after transection
D
Repair genes Cl7
log2 expression ratio relative to iSC
Days after transection

Figure S2. Related to Figure 2

Differentially expressed genes in bSC and dSC

(A) Volcano plot of DESeq2 differential gene expression analysis between bridge (bSC) and distal (dSC) Schwann cell RNA-seq datasets. Genes regulated over 2.5 fold (adjusted $p < 0.05$) are highlighted in light blue.

(B) Representative images of longitudinal cryo-sections of transected *tdTom*^{SC} nerves collected at days 4, 6, 8 and 10 post-transection. Schwann cells are shown in red and nuclei are counterstained with DAPI. Dotted lines indicate the boundaries of the bridge region, P denoted the proximal and D distal stumps at each time point. Note that migration of Schwann cells into the wound begins at day 4, peaks at d6 and subsides by day 8, when Schwann cell cords from proximal and distal stumps join together to reconnect the severed nerve ends.

(C, D) RNA-seq time course analysis of distal (dSCs) or bridge (bSCs) Schwann cell gene expression changes after sciatic nerve transection. dSCs/iSCs (blue) or bSCs/iSCs (red). DESeq2 expression ratios are plotted as a function of time after injury for two groups of repair genes associated with inflammation and ECM production, identified in cluster 1 (Cl1, C) and cluster 7 (Cl7, D) of figure 2A. Boxes denote the interquartile range and black strikes the median of all ratios in the list. Open boxes represent dSCs/iSCs expression ratios for all genes as a reference. Note that the repair genes expression is the highest in dSCs from day 6 onwards coinciding with robust SC invasion into the bridge. Data were acquired for cells isolated from single nerves, n=3-4 per time point.

Figure S3

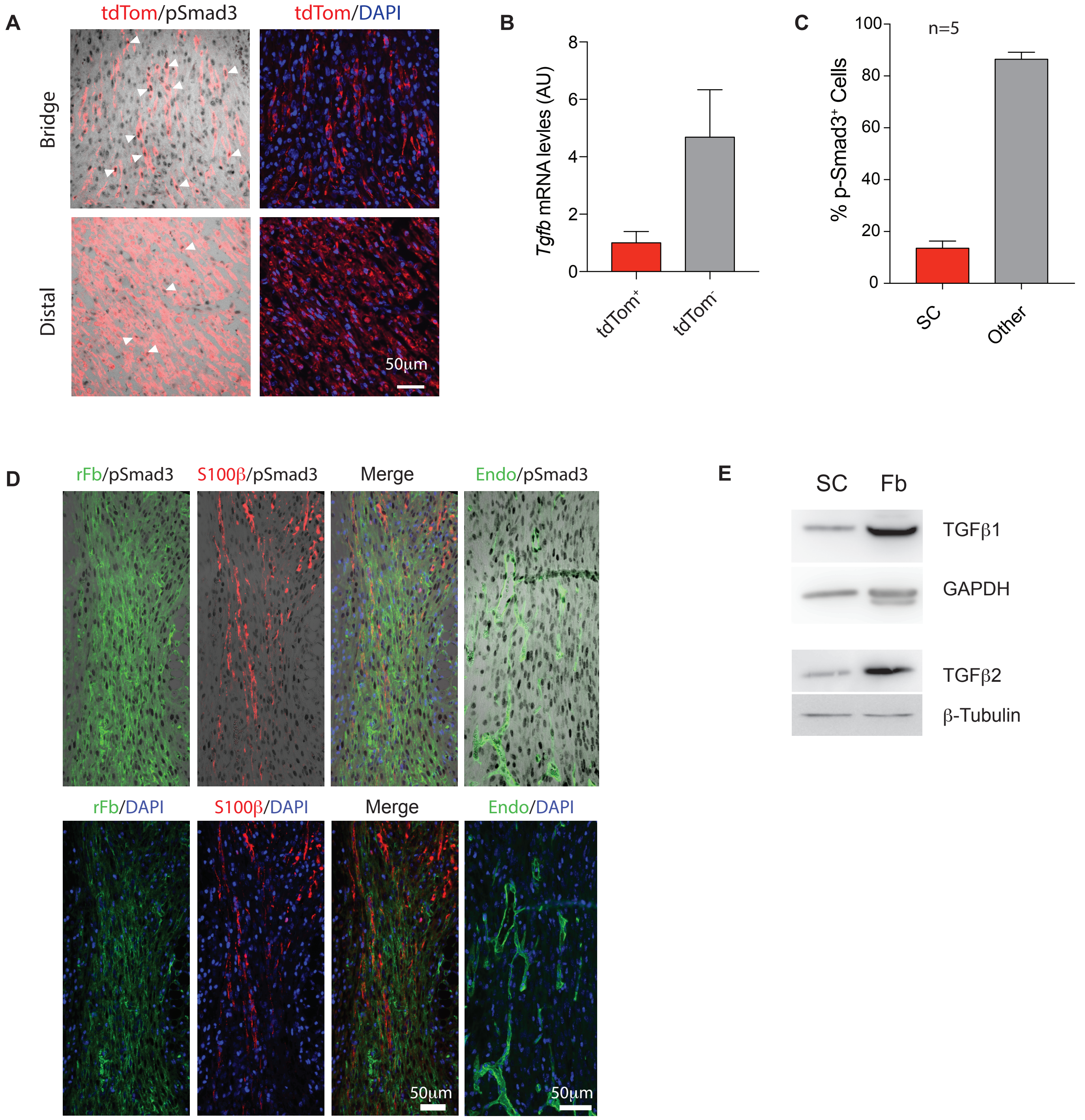
A
tdTom/pSmad3
tdTom/DAPI
Bridge
Distal
50μm
B
Tgfb mRNA levles (AU)
8
6
4
2
0
tdTom+
tdTom-
C
% p-Smad3+ Cells
n=5
100
80
60
40
20
0
SC
Other
D
rFb/pSmad3
S100β/pSmad3
Merge
Endo/pSmad3
rFb/DAPI
S100β/DAPI
Merge
Endo/DAPI
50μm
50μm
E
SC
Fb
TGFβ1
GAPDH
TGFβ2
β-Tubulin

Figure S3. Related to Figure 3

TGFβ expression is enriched in the bridge microenvironment

(A) p-Smad3 expression in the bridge (top) and distal (bottom) regions of cut $tdTom^{SC}$ nerves 6 days post-transection. Paraffin sections were stained for p-Smad3 (DAB), tdTomato (red) and DAPI (blue). White arrow heads indicate p-Smad3 co-localisation with SC nuclei. Numbers of p-Smad3$^+$ Schwann cells were similar to those found in Figure 3A and B using S100β.

(B) Quantitative RT–PCR analysis of TGFβ expression in FACS-purified tdTomato$^-$ bridge cells relative to tdTomato$^+$ bridge Schwann cells. Data are represented as mean ±SEM, n=3.

(C) Quantification of the numbers of p-smad3$^+$ Schwann cells and other nerve cells in the bridge at 6 days post-transection assessed by immunohistochemistry as in Figure 3A. Approximately 50% of the total number of bridge cells express p-smad3. Of these cells, approximately 15-20% are Schwann cells and 80-85% are other wound cells. Data are represented as mean ± SEM, n=5.

(D) Characterisation of p-smad3$^+$ cell types in the bridge. Representative images of paraffin sectioned nerves collected 6 days post-transection which were used for the quantifications presented in (B). Sections were stained for p-smad3 (DAB; top panel) and a fibroblast marker (reticular fibroblast rFb, green) to assess TGFR activity in fibroblasts and for the endothelial marker endomucin (green, right panel) to assess activity in blood vessels. Schwann cells were also labelled with S100β (red) and nuclei counterstained with DAPI (blue). Note that the majority of p-Smad3$^+$ non-Schwann bridge cells are fibroblasts and endothelial cells.

(E) Western blot analysis of TGFβ1 and TGFβ2 levels in cultured rat Schwann cells (SC) and perineurial fibroblasts (Fb). GAPDH and β-tubulin served as loading control.

Clements et al. Figure S4

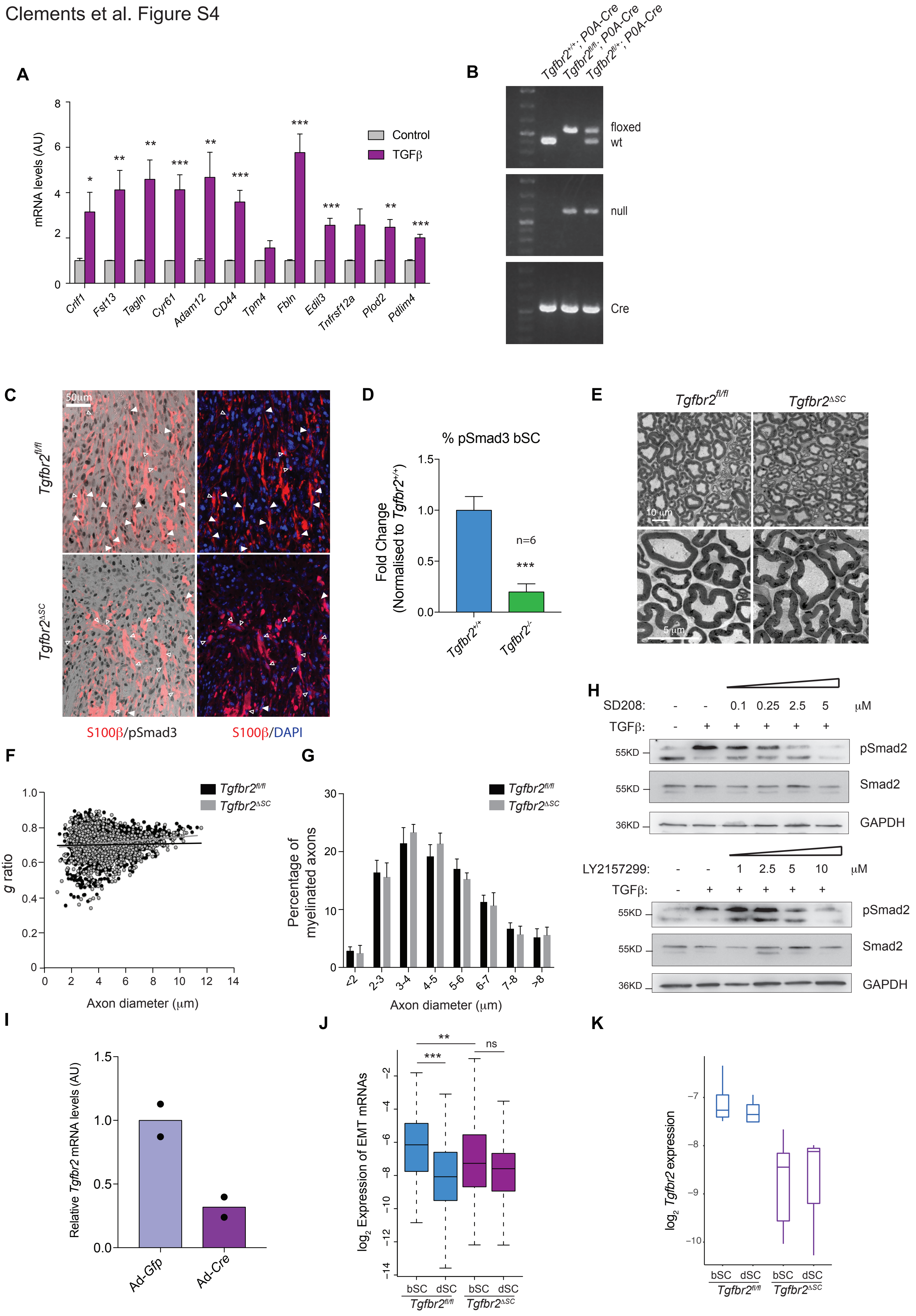

Figure S4. Related to Figure 4

Efficiency of *Tgfbr2* recombination in *Tgfbr2*$^{\Delta SC}$ mice

(A) Quantitative RT–PCR analysis of a panel of EMT genes that are specifically increased in bridge cells carried out in cultured rat Schwann cells untreated or treated with TGFβ for 16h. Data are represented as mean ± SEM,n=3. *P* values were calculated using Students t-test *p<0.05; **p<0.01; ***p<0.001.

(B) PCR analysis of genomic DNA for floxed, wild type and recombined *Tgfbr2* allele and for P0A-Cre transgene. DNA was extracted from sciatic nerves of *Tgfbr2*$^{fl/fl}$, *Tgfr2*$^{fl/-}$; *POA-Cre* and *Tgfbr2*$^{\Delta SC}$ mice, as indicated.

(C) p-Smad3 expression in the nerve bridge of *Tgfbr2*$^{fl/fl}$ and *Tgfbr2*$^{\Delta SC}$ mice collected at 6d post-transection. Paraffin sections were stained for p-Smad3 (DAB), S100β (red) and DAPI (blue).

(D) Quantification of the numbers of pSmad3^{+} *Tgfbr2*$^{fl/fl}$ and *Tgfbr2*$^{\Delta SC}$ bridge Schwann cells depicted in C. p-Smad3 expression is strongly reduced in the Schwann cells of knock-out animals relative to wild-type controls. n=10 for *Tgfbr2*$^{fl/fl}$ and 6 for *Tgfbr2*$^{\Delta SC}$ animals. Data are normalised to *Tgfbr2*$^{fl/fl}$ controls and represent mean ± SEM. *P* values were calculated using the two-tailed Student t-test n=5. ***p<0.001.

(E) Semithin sections (top panels) and electron micrographs (bottom panels) of *Tgfbr2*$^{fl/fl}$ and *Tgbfr2*$^{\Delta SC}$ adult nerves. Scale bars, 10μm (top panels) and 5μm (bottom panels). Deletion of *Tgfbr2* in Schwann cells does not alter nerve structure.

(F) *g* ratios expressed as a function of axon diameter do not vary between intact nerves of *Tgfbr2*$^{fl/fl}$ (black dots) and *Tgfbr2*$^{\Delta SC}$ (grey dots) mice. The graph represents the *g* ratios obtained from more than 650 myelinated axons per genotype. n=3 per group.

(G) Quantification of distribution of myelinated fibers in intact *Tgfrb2*$^{fl/fl}$ and *Tgfbr2*$^{\Delta SC}$ nerves. No significant difference in myelination is observed. Data are represented as mean ± SEM n=4; *Tgfbr2*$^{fl/fl}$ (black bars) *Tgfbr2*$^{\Delta SC}$ (grey bars).

(H) Western blot analysis of p-Smad2 and total Smad2 in rat Schwann cells left untreated or treated with TGFβ in the absence or presence of different concentration of TGFR inhibitors, as indicated. Note complete suppression of TGFR signalling at the concentrations used in this study (2.5mM for SD208 top; 5mM for LY2157299 bottom). GAPDH served as loading control.

(I) qRT-PCR analysis of *Tgbfr2* mRNA levels in cultured mouse Schwann cells prepared from *Tgfbr2*$^{fl/fl}$ mice and transduced with adenoviral GFP-Cre recombinase (adCre) to induce *Tgfbr2* recombination or adentoviral GFP (adGFP) as control. n=2.

(J) qRT-PCR analysis of 24 bridge-specific EMT genes in FACS-purified bridge (bSC) and distal (dSC) Schwann cells from the bridge and distal regions of single nerves of *Tgfbr2*$^{fl/fl}$ mice (blue boxes, n=6) and *Tgfbr2*$^{\Delta SC}$ (purple boxes, n=5) at 6 days post-transection. Deletion of *Tgfbr2* in Scwhwann cells impairs the induction of EMT genes in the bridge, but not in the distal stump 6 days post-transection. Data are represented as $\log_2$ expression of all EMT genes and are the same as those used on figure 4F. The whiskers extend to the most extreme data point which is no more than 1.5 times the interquartile range from the box. ** $p<0.01$, ***$p<0.001$.

(K) qRT-PCR of *Tgfbr2* expression in FACS-purified bridge (bSC) and distal (dSC) from *tdTom;Tgfbr2*$^{fl/fl}$ (blue boxes, n=6) and *tdTom;Tgfbr2*$^{\Delta SC}$ (purple boxes, n=5) mice 6 days post-transection. As expected, *Tgfbr2* levels are downregulated in tdTom;*Tgfbr2*$^{\Delta SC}$. Variability in the extent of *Tgfbr2*mRNA reduction was observed, consistent with the detected variability in recombination efficiency (Figure S4D). The whiskers extend to the most extreme data point which is no more than 1.5 times the interquartile range from the box.

Figure S5

A

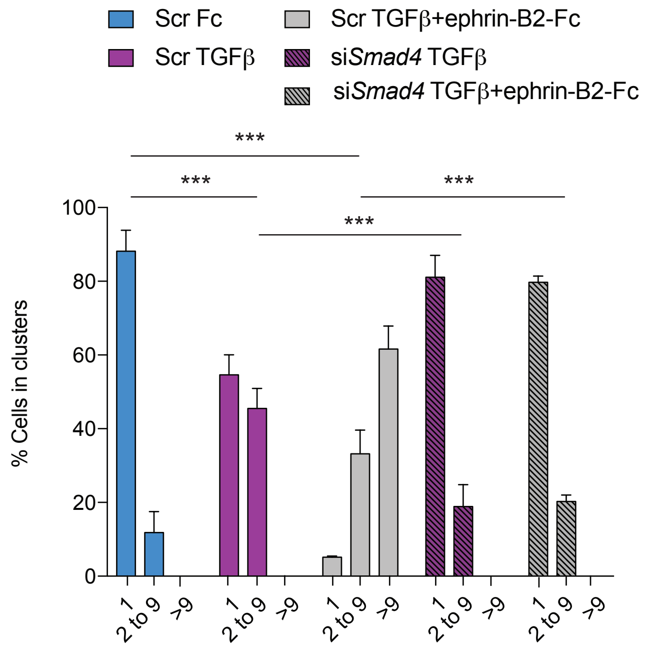

B

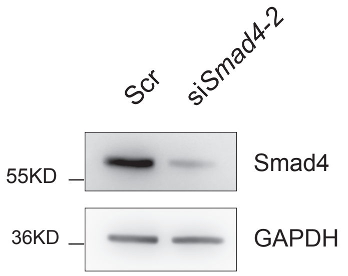

C

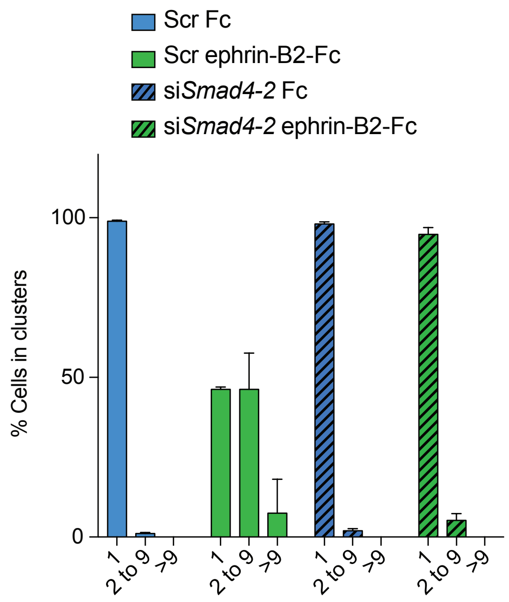

Figure S5. Related to Figure 5

Efficacy and specificity of Smad4 knock-down

(A) Quantification of clustering of Scr and *Smad4*-siRNA treated Schwann cells plated on Fc control proteins or ephrin-B2-Fc ligands in the presence or absence of TGFβ, as indicated. TGFβ effects were completely abrogated in *Smad4* knock down cells, confirming efficacy of pathway inactivation. Cell clustering data is represented as mean ± SEM. n=3, ***$p<0.001$, Fisher's exact test.

(B) Western blot analysis of *Smad4* levels in Schwann cells treated with Scr oligos or a second independent siRNA oligo to *Smad4* (*Smad4-2*). β-actin served as a loading control.

(C) Quantification of clustering of Scr and *Smad4-2* siRNA treated Schwann cells plated on Fc control proteins or ephrin-B2-Fc ligands, as indicated. n=2. Cell clustering data is represented as mean ± SEM.

Figure S6

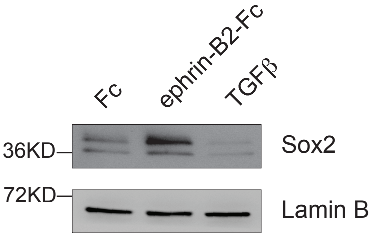

Figure S6. Related to Figure 6

TGFβ does not cross-talk with EphB2 via Sox2

Western blot analysis of Sox2 levels in nuclear extracts of rat Schwann treated with Fc, ephrin-B2-Fc or TGFβ for 16h. Lamin B was used as loading control. Note that TGFβ does not increase Sox2 levels.

Figure S7

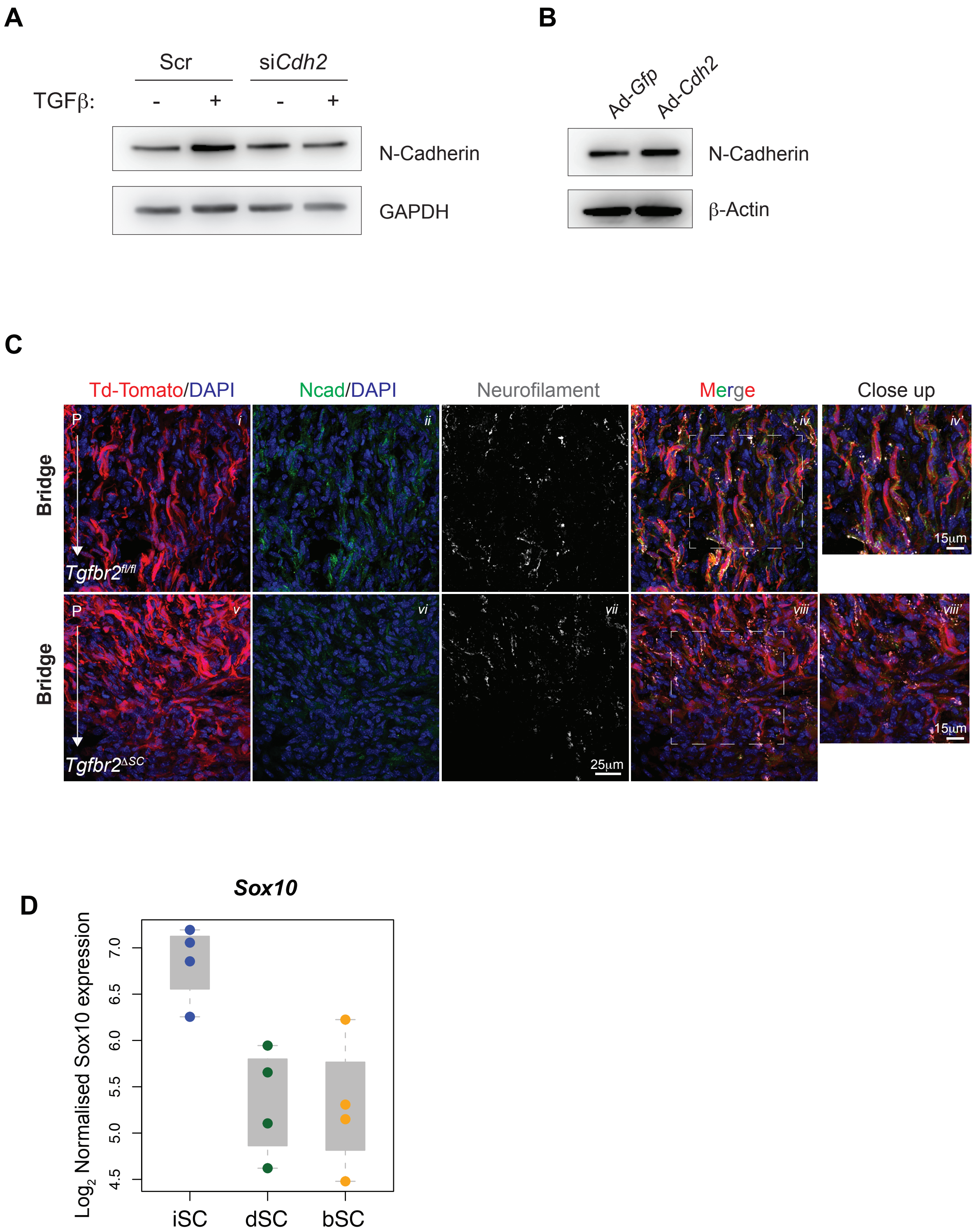

A
Scr
siCdh2
TGFβ:
-
+
-
+
N-Cadherin
GAPDH
B
Ad-Gfp
Ad-Cdh2
N-Cadherin
β-Actin
C
Td-Tomato/DAPI
Ncad/DAPI
Neurofilament
Merge
Close up
Bridge
P
Tgfbr2fl/fl
Tgfbr2ΔSC
15µm
25µm
D
Sox10
Log2 Normalised Sox10 expression
4.5
5.0
5.5
6.0
6.5
7.0
iSC
dSC
bSC

Figure S7. Related to Figure 7

Manipulation of N-cadherin levels for gain and loss of function studies

(A) Western blot analysis of N-cadherin levels in Scr-treated and partial *Cdh2* knock-down (*siCdh2*) Schwann cells before and after TGFβ treatment. GAPDH is used as loading control. Note that partial knock-down does not significantly affect basal N-cadherin levels but fully blocks the TGFβ-induced increase in N-cadherin observed in control Scr cultures.

(B) Western blot analysis of N-cadherin levels in Schwann cells transduced with GFP (Ad-*Gfp*) or *Cdh2* (Ad-*Cdh2*) encoding adenoviruses. β-actin served as loading control. The titer used for infection increased N-cadherin levels by approximately two-fold, a similar extent as TGFβ treatment in control cells (see Figure 6I).

(C) Representative images of N-cadherin staining in nerve bridges from tdTom;$Tgfbr2^{fl/fl}$ (top panels, *i-iv'*) and $Tgfbr2^{\Delta SC}$ (bottom panels, *v-viii'*) mice 6d post-transection. Sections were stained for N-cadherin (green) and neurofilament (grey). Note the minimal co-localisation between N-cadherin and neurofilament and similar axonal denisties in both genotypes, indicating that the differences in N-cadherin expression are not caused by the delayed axonal regrowth of $Tgfbr2^{\Delta SC}$ mice. Inset panels are higher magnification of merged images *iv* and *viii* delinated by the dashed line. Scale bar = 25µm *i-iv* and *v-viii* and 10µm in close up panels.

(D) Boxplot of RNA-seq FPKM expression scores for *Sox10* in intact SCs (iSC, blue), bridge SCs (bSC, yellow) and distal SCs (dSC, green). Coloured dots represent single biological repeats. n=4, p=0.014 for both bSC and dSC relative to iSC. The whiskers extend to the most extreme data point which is no more than 1.5 times the interquartile range from the box.
